# Versatile unsupervised design of antennas using flexible parameterization and computational intelligence methods

**DOI:** 10.1038/s41598-024-80319-z

**Published:** 2024-11-29

**Authors:** Slawomir Koziel, Anna Pietrenko-Dabrowska, Stanislaw Szczepanski

**Affiliations:** 1 Engineering Optimization & Modeling Center, Reykjavik University, 102 Reykjavik, Iceland; 2 Faculty of Electronics, Telecommunications and Informatics, Gdansk University of Technology, Gdansk, 80-233 Poland

**Keywords:** Unsupervised design, Antenna parameterization, Machine learning, Design automation, Bio-inspired optimization, Engineering, Electrical and electronic engineering

## Abstract

Developing contemporary antennas is a challenging endeavor that requires considerable engineering insight. The most laborious stage is to devise an antenna architecture that delivers the required functionalities, e.g., multiband operation. Iterative by nature (hands-on topology modifications, parametric studies, trial-and-error geometry selection), it typically takes many weeks and requires considerable engagement from a human expert. Consequently, only a few possible design options concerning the fundamental antenna geometry may be considered. Automated topology rendition and geometry parameter optimization are highly relevant, especially from the industrial perspective. Therein, reducing time-to-market and limiting the involvement of trained experts is critical. This research proposes an innovative procedure for unsupervised development of planar antennas. Our method leverages flexible antenna parameterization based on re-sizable elliptical patches. It permits the realization of a massive number of geometries of diverse shapes and complexities using a small number of decision variables. Computational intelligence methods are employed to conduct antenna evolution exclusively based on specifications and possible constraints (e.g., maximum size). Fine-tuning of the structure geometry is achieved through low-cost local search routines. Our methodology is demonstrated by designing several antennas featuring distinct characteristics (broadband, single-, dual- and triple-band). The obtained results, supported by experimental data, underscore the presented approach’s versatility and capability to render unconventional topologies at reasonably low computational expenses. As mentioned earlier, the design process is fully automated without human expert involvement.

## Introduction

Antennas are the fundamental elements of wireless communication systems (mobile phones, satellite communication, internet of things, etc^1–4^). , and other technologies such as radio-frequency identification, ambient energy harvesting, wearable/implantable electronics, medical imaging, to name just a few^5–8^. Conventional design approaches typically begin with existing antenna geometries available in previous generations of products, scientific publications, or structures developed using engineer’s insight. Subsequently, modifications are made to achieve the required functionality (e.g., circular polarization, multi-band operation). Having a rough design, parametric studies are employed^9,10^, or, recently, rigorous optimization methods such as bio-inspired techniques enhanced by the response-feature technology^11^ or multi-objective Bayesian optimization^12^ to adjust antenna dimensions, thereby boosting its performance concerning the figures of interest (impedance matching, gain, axial ratio). Most development stages, including optimization, use full-wave electromagnetic (EM) simulation models^13^ for evaluation dependability. The literature is replete with optimization strategies, specialized in local (e.g., sensitivity-based, derivative-free^14–18^), global^19–29^ and multi-objective design^30–34^, and uncertainty quantification (UQ)^35–38^. Local methods incorporate various gradient-based algorithms^14^ also based on dedicated fast solvers^15^, procedures with sparse sensitivity updating schemes^16,17^, or stencil-based methodologies (i.e., various types of pattern search methods^18^). Global approaches often employ nature-inspired routines such as Grey Wolf Optimizer (GWO)^19^ or particle swarm optimization^20^, but also diverse machine learning methods^22^, and surrogate-assisted algorithms using both forward models (e.g., neural networks^23^, kriging^24^) and inverse ones^21^. On the other hand, multi-objective optimization is typically involving surrogate models (e.g., kriging)^30^, and incorporating mechanisms such as dimensionality reduction^31^ or objective aggregation^32^. UQ is heavily based on fast replacement models^36^ often specialized ones such as polynomial chaos expansion (PCE)^37^. Numerous accelerated techniques were developed as EM-driven design exerts significant computational costs^35^. Some worth mentioning methods include already mentioned surrogate-based routines, which can be based on data-driven metamodels (e.g., kriging^36,38^, PCE^37,39^, diverse types of artificial neural networks^38,41,42–45,46^, sometimes enhanced by other technique such as response features^40^. On the other hand, physics-based surrogates are also utilized, such as space mapping^47,48^ or a combination thereof with neural networks^49^. A popular method for accelerating EM-driven design is machine learning (ML)^50,51^, where the surrogate model (e.g., neural network^52,53^, deep neural network^54^) is used as a predictor yielding presumably high-quality candidate designs is iteratively updated using accumulated EM data^55,56^. Other popular techniques include the response feature technology^57^, exploiting a specific structure of the system’s outputs and weakly nonlinear dependence of the characteristic points on design variables^58^, often in connection with other methods (e.g., space mapping^59^ and multi-fidelity models^60^). Yet another approach is variable-resolution methods^61^, where the design process is expedited by incorporating faster but less accurate coarse-discretization models (or even equivalent circuit representations)^62,63^. To be reliably used in the design process, the lower-fidelity models are typically corrected using sparsely sampled high-fidelity data^64^ or utilized to carry out selected operations within the optimization framework^65^. A recent review of model- and ML-based antenna design methods can be found in^66^.

Performance enhancement and implementing additional functionality is often realized by adjusting basic antenna geometries (patches, monopoles, etc.), depending on the designer’s experience and preferences. Geometry optimization typically leads to designs that resemble the initial ones^67,68^. This and the long time required for experimentation with any given architecture are serious limiting factors regarding the number of alternative topologies that might be considered as potentially better options for a given application. Topology optimization (TO) is a different approach that allows for the adjustment of antenna geometry. One possibility is the spatial discretization of the area assigned to the antenna into pixels (square or rectangular shape), which may be filled with metal or left empty^69–73^. For example, in^69^, a radiator part of the antenna is discretized in the abovementioned manner, whereas in^70^, a discretized surface is employed to generate a high-performance metalens antenna. The work^71^ presents a subwavelength planar monopole antenna designed through the evolutionary generation of metalization patterns of the antenna’s radiator. Similar idea is exploited in^73^ to create a rectangular horn antenna. The pixel arrangement is typically determined using computational intelligence methods, primarily genetic algorithms (e.g.,^74,75,80^ binary particle swarm optimizer (BPSO)^76^, or quantum genetic algorithm^77^. In all these cases, due to the discretized structure of the antenna, the applied bio-inspired methods must be adopted to handle the combinatorial nature of the underlying optimization task. These techniques improve design flexibility at the expense of turning antenna development into a combinatorial problem of high complexity. In some approaches, only a specific antenna part (e.g., the radiator) is discretized^69^. Pixel antennas are a somehow different approach where the structure geometry is decided by allocating connections between pre-defined unit cells (typically squares). In some cases, the arrangement of the building block connections is realized using bio-inspired metaheuristic algorithms^78,79^. In other cases, multi-objective evolutionary algorithms are used^82^, or even gradient-based optimization incorporating adjoint sensitivities^81,83^. Yet another option is free-form TO, which enables considerably improved flexibility as the antenna geometry may take almost any shape^84–89^. For example, in^84^ an isolation structure in MIMO antenna has been developed through TO, whereas in^85^ topology optimization has been used to design a conical-beam antennas. The reference^86^ presents design of sub-wavelength antenna using TO. The design of antennas for energy harvesting can be found in^87^. Meanwhile, in^88^, TO was employed to develop both linear- and circularly polarized patch antenna structures. Finally^89^, presents the optimization of a multi-layer metasurface using a combination of TO and inverse neural network models. Free-form TO methods often employ fast custom-designed EM solvers to accelerate the development process^15,90–94^. This is necessary because the underlying optimization tasks are large-scale, so fast antenna evaluation is essential to make the TO-based methods practical. For example, the work^90^ employs a custom-designed finite-difference time-domain (FDTD) solver, similar to^91 and94^, whereas in^92^, a fast method of moments has been presented. A disadvantage of free-form TO is that the optimization uses gradient-based algorithms, making the outcome dependent on the initial architecture. Also, these techniques cannot be integrated with commercial simulation software packages, which is another limiting factor from the engineering perspective.

Considering the deficiencies of the above methods, a procedure allowing simultaneous determination of antenna geometry, and its optimum dimensions would be of considerable practical value. The crucial prerequisites include sufficient flexibility (in terms of an extensive range of architectural variations), the capability of implementing structures of diverse characteristics (broadband, multiband), and the possibility to integrate with commercial EM solvers. These features are essential for industrial applications from the perspective of time-to-market and the ability to devise unconventional antenna topologies potentially better suited for highly functional devices. This research attempts to deliver an innovative procedure that exhibits the mentioned properties and allows for automated specification-driven planar antenna development. The presented methodology capitalizes on the flexible parameterization of the antenna geometry involving elliptical patches and gaps of adjustable position and sizes. This allows for the implementation of a massive number of topologies of various shapes and complexity while using a limited set of decision variables. The design process is unsupervised and employs a combination of computational intelligence techniques to generate devices that fulfill the specifications concerning reflection responses and optional constraints (such as maximum size). Final geometry parameter tuning is done using local (gradient-based) algorithms. The specific dimensions and topology may be altered to boost the system’s performance at this stage. For demonstration, our technique is applied to design several antenna structures operating at different frequency bands and offering various functionalities (broadband, single-, dual- and triple-band). The results corroborate the versatility of our method and underscore its capability to produce unconventional topologies at practically acceptable computational expenses. The design process is utterly unsupervised, and no human-expert interaction is required.

This study delivers several technical contributions, which include (i) a novel and versatile antenna parameterization for generating immense variety of geometries with limited number of design variables, (ii) the development of automated design procedure combining bio-inspired and conventional (gradient-based) algorithms, (iii) demonstrating the capability of our method to conduct unsupervised antenna evolution and dimension tuning, (iv) demonstrating versatility of the framework through the design of structures featuring diverse functionalities obtained through purely specification-driven algorithm execution with no setup adjustments whatsoever, (v) ensuring that the presented approach can work in synergy with commercial EM simulation packages, thereby making it suitable for academic and industrial applications. Given the listed properties, the suggested algorithm can be considered an interesting and practical alternative to existing antenna design automation methodologies.

## Specification-driven antenna design: the algorithm

This part of the study explains the details of the proposed automated development procedure. The considered type of antennas are planar structures implemented on single-layer dielectric substrates. Section 2.1 discusses antenna parameterization and demonstrates its flexibility. Section 2.2 and 2.3 outline the computational model and the algorithmic tools utilized to carry out the evolution and dimension adjustment of the antenna structure, respectively. The complete framework is summarized in Sect. 2.4.

### Antenna parameterization

The fundamental component of the unsupervised design framework is antenna parameterization. Its essential features are simplicity (for easy handling), flexibility (to allow a multitude of distinct geometries, e.g., monopoles, dipoles, and patch antennas), and a limited set of design variables. The latter is essential to make the antenna evolution and optimization process numerically tractable and keep the computational costs practically acceptable. The design variables should be continuous to permit gradient-based tuning and discrete parameters to adjust the structure’s complexity as necessary. Furthermore, the parameterization must be straightforward to implement within commercial EM simulation environments (here, CST Microwave Studio is used as an underlying EM solver).

The components of the proposed parameterization that comply with the mentioned requirements have been listed in Table 1. In this study, the antenna is assumed to be rectangular, with an adjustable-length ground plane, discrete port, and several patches and gaps of adjustable location and size. The number *N*_*P*_ of patches and *N*_*G*_ of gaps determine the antenna complexity and can be treated as auxiliary variables during the antenna development. Another option is to decide about the complexity upfront and only employ continuous parameters, which will be employed when demonstrating the procedure in Sect. 3. For convenience, the position and sizes of all components relative to the substrate width *W* and length *L*. They are recalculated into absolute (physical) values for EM analysis.

**Table 1:** Components of flexible planar antenna parameterization.

Component	Graphical Illustration	Parameters		Comments
		Relative	Absolute	
Substrate		–	*W* – width *L* – length	Rectangular substrate is assumed
Discrete port		*P*_*x.r*_ – horizontal position *P*_*y.r*_ – vertical position	*P*_*x*_ = *P*_*x.r*_*W*/2 *P*_*y*_ = *P*_*y.r*_*L*/2	Port position is determined relative to the substrate center
Ground plane		*L*_*g.r*_ – ground plane length	*L*_*g*_ = *L*_*g.r*_*L*/2	Ground plane length is relative to the substrate length
Elliptic patch		*S*_*x.i.r*_ – horizontal position of *i*th patch *S*_*x.i.r*_ – vercical position of *i*th patch *a*_*x.i.r*_ – horizontal axis of *i*th patch *a*_*y.i.r*_ – vertical axis of *i*th patch	*S*_*x.i*_ = *S*_*x.i.r*_*W*/2 *S*_*y.i*_ = *S*_*y.i.r*_*L*/2 *a*_*x.i*_ = *a*_*x.i.r*_*W*/2 *a*_*y.i*_ = *a*_*y.i.r*_*L*/2	Patch size is relative to the substrate size
Elliptic gap		*R*_*x.i.r*_ – horizontal position of *i*th gap *R*_*x.i.r*_ – vercical position of *i*th gap *b*_*x.i.r*_ – horizontal axis of *i*th gap *b*_*y.i.r*_ – vertical axis of *i*th gap	*R*_*x.i*_ = *R*_*x.i.r*_*W*/2 *R*_*y.i*_ = *R*_*y.i.r*_*L*/2 *b*_*x.i*_ = *b*_*x.i.r*_*W*/2 *b*_*y.i*_ = *b*_*y.i.r*_*L*/2	Gap size is relative to the substrate size

**Fig. 1:**
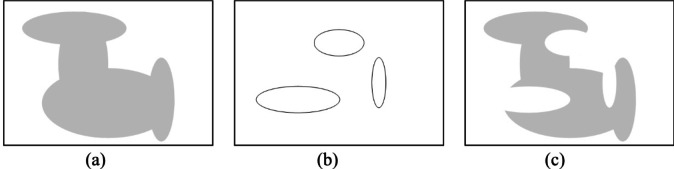
Assembling antenna geometry (front metallization only): (**a**) combined elliptic patches, (**b**) location of gaps, (**c**) complete geometry obtain by Boolean-wise subtracting metallization at the gaps.

**Fig. 2:**
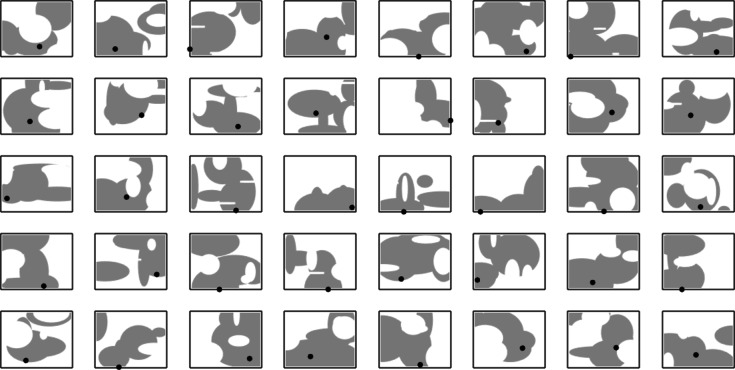
Flexibility of the proposed antenna parameterization demonstrated using random architectures generated assuming *N*_*P*_ = 5 and *N*_*G*_ = 2. Front-side metallization is shown (gray), along with the location of the discrete port (black dot).

The parameters listed in Table 1 are aggregated into a single vector that will be handled by the optimization procedures outlined in the next section. Given *N*_*P*_ and *N*_*G*_, this takes the form of

1 $${\varvec{x}}={\left[ {\begin{array}{*{20}{c}} {LW{P_{x.r}}{P_{y.r}}{L_g}{S_{x.1.r}}{S_{y.1.r}}{a_{x.1.r}}{a_{y.1.r}} \ldots {S_{x.{N_p}.r}}{S_{y.{N_p}.r}}{a_{x.{N_p}.r}}{a_{x.{N_p}.r}} \ldots } \\ {{R_{x.1.r}}{R_{y.1.r}}{b_{x.1.r}}{b_{y.1.r}} \ldots {R_{x.{N_G}.r}}{R_{y.{N_G}.r}}{b_{x.{N_G}.r}}{b_{y.{N_G}.r}}} \end{array}} \right]^T}$$

Total number of design variables is *n* = 5 + 4(*N*_P_ + *N*_*G*_).

Figure 1 shows assembling the antenna geometry by concatenating the elliptical patches and etching out the gaps for an exemplary setup. As indicated in Fig. 2, our parameterization enables a large variety of distinct topologies, even with a relatively small number of parameters (here, shown for *N*_*P*_ = 5 and *N*_*G*_ = 3). These shapes cannot be generated using traditional methods. Further, as the position and sizes of the building blocks are continuous variables, the antenna architecture may undergo global evolution and local tuning. For specific application areas, the antenna size may also be the subject of optimization or setup fixed to any specific substrate width and length values. This is a significant advantage over both pixel antennas and free-form topology optimization. Removing specific components from the computational model is realized by assigning them zero size.

### Computational model

The computational representation of the designed device is prepared in CST Microwave Studio^95^. Table 1 lists all components of the proposed model. The pre-implemented number of patches and gaps is ten and six, respectively, which is more than sufficient for practical applications. As mentioned earlier, if the actual number of building blocks is set to smaller values, the redundant ones are assigned zero size, effectively being disabled.

**Fig. 3:**
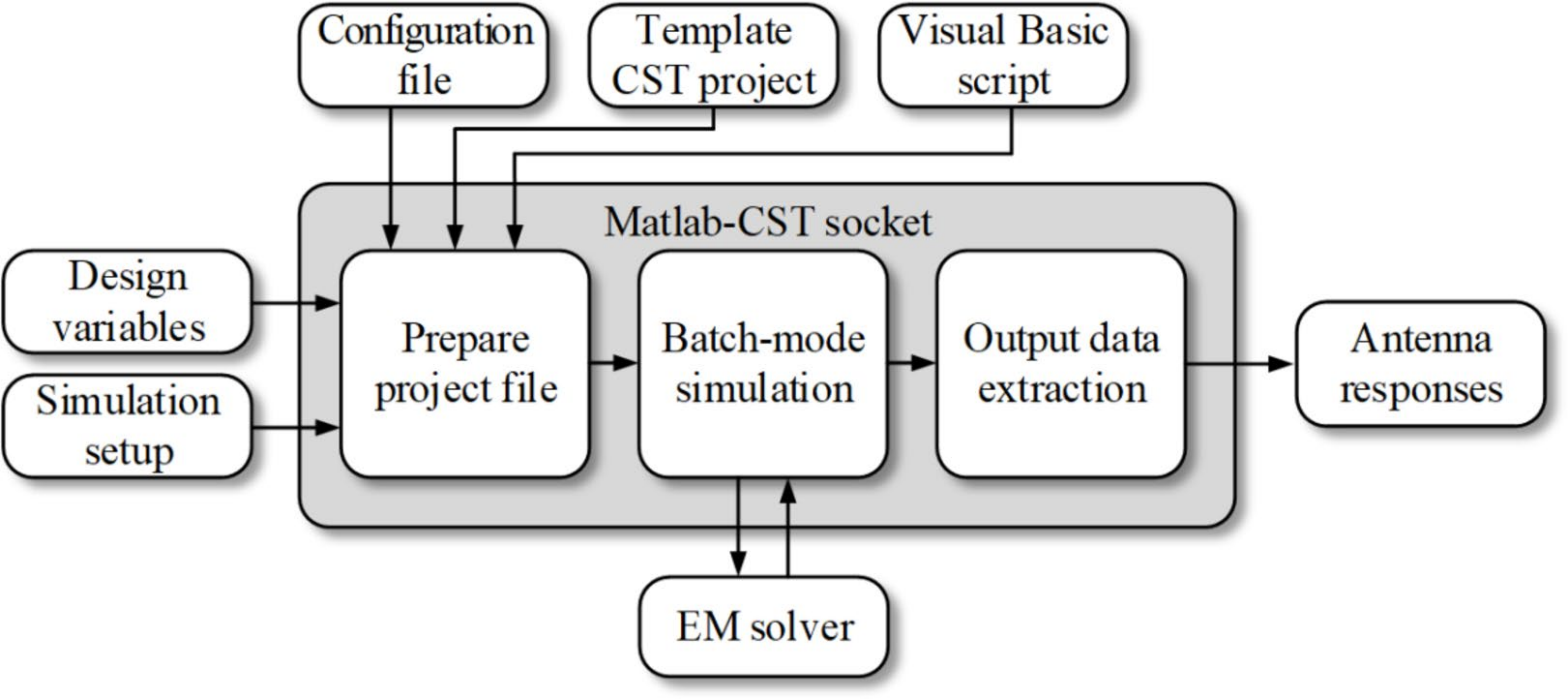
Operating flow of antenna response evaluation. Design variables and simulation setup are used along with the computational model templates to prepare the project file. After the batch-mode EM analysis, antenna characteristics are extracted from the exported simulation data.

To evaluate antenna characteristics, the design variable vector ***x*** is recalculated from relative to absolute parameters (cf. Table 1). The excessive patch metallization is trimmed to the substrate size. EM simulation is carried out in a batch mode with the antenna parameters controlled through a Visual Basic script. Upon simulation, the antenna responses are extracted from the output files exported by CST. The operating flow of the EM model evaluation scheme using the abovementioned concepts is presented in Fig. 3.

### Optimization-driven antenna development

Here, we formulate the design task and discuss the architecture development of the antenna using computational intelligence methods and its final tuning through gradient-based optimization.

#### Design task

The design problem is posed for antenna impedance matching to make sure that the in-band |*S*_11_| ≤ − 10 dB over the band of interest *F*. For multi-band antennas, *F* = [*f*_1_ – *B*_1_/2, *f*_1_ + *B*_1_/2] ∪ [*f*_2_ – *B*_2_/2, *f*_2_ + *B*_2_/2] ∪ … ∪ [*f*_*K*_ – *B*_*K*_/2, *f*_*K*_ + *B*_*K*_/2], where *f*_*k*_ and *B*_*k*_ are the center frequencies and the respective bandwidths (*K* being the number of bands). Consequently, our objective is to identify the parameter vector x^*^

2 $${{\varvec{x}}^*}=\arg \mathop {\hbox{min} }\limits_{{{\varvec{x}} \in X}} U\left( {\varvec{x}} \right)$$

with

3 $$U\left( {\varvec{x}} \right)=\mathop {\hbox{max} }\limits_{{f \in F}} \left\{ {\left| {{S_{11}}\left( {{\varvec{x}},f} \right)} \right|} \right\}$$

In (2) *X* is the design space delimited by the bounds on geometry parameters. Recall that the parameterization proposed in Sect. 2.1 enables the vector ***x*** to represent both the antenna architecture and dimensions. Consequently, solving the problem (2) simultaneously adjusts the geometry and improves impedance matching performance. Additional requirements might also be imposed (e.g., miniaturization, gain, and/or radiation pattern requirement), which will be considered elsewhere.

It should be noted that in the considered case, a minimax objective function (3) is employed, which improves the impedance matching over the target frequency ranges. This is the formulation used in the result section (Sect. 3) of this work. Other formulations are possible, for example, targeting the improvement of the antenna gain, reducing its footprint (when using the substrate’s width and length are additional design variables), etc. In this paper, we focus on perhaps the most widely addressed objective which is impedance matching. Other design scenarios will be considered elsewhere.

Similarly, it is possible to make the parameterization even more flexible by incorporating additional parameters such as elliptical patches and gaps for the ground plane (rather than using a simple rectangular ground). This and other options will be considered in future work.

#### Antenna topology evolution

**Fig. 4:**
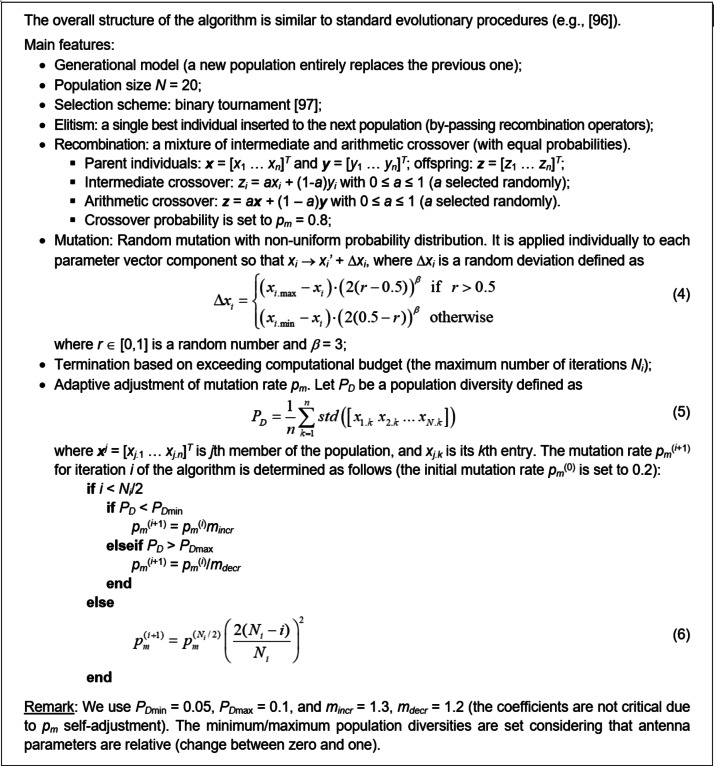
The evolutionary algorithm used to carry out the evolution of antenna topology.

The first stage of antenna development is simultaneous global optimization of the architecture (spatial allocation of the patches and gaps) and the adjustment of building block dimensions. This is realized using a floating-point evolutionary algorithm ^96,97^ that incorporates elitism and adaptive mutation rate, outlined in Fig. 4. It should be noted that *p*_*m*_ gradually decreases to zero later in the optimization process, improving the algorithm’s exploitation capability.

#### Local tuning

**Fig. 5:**
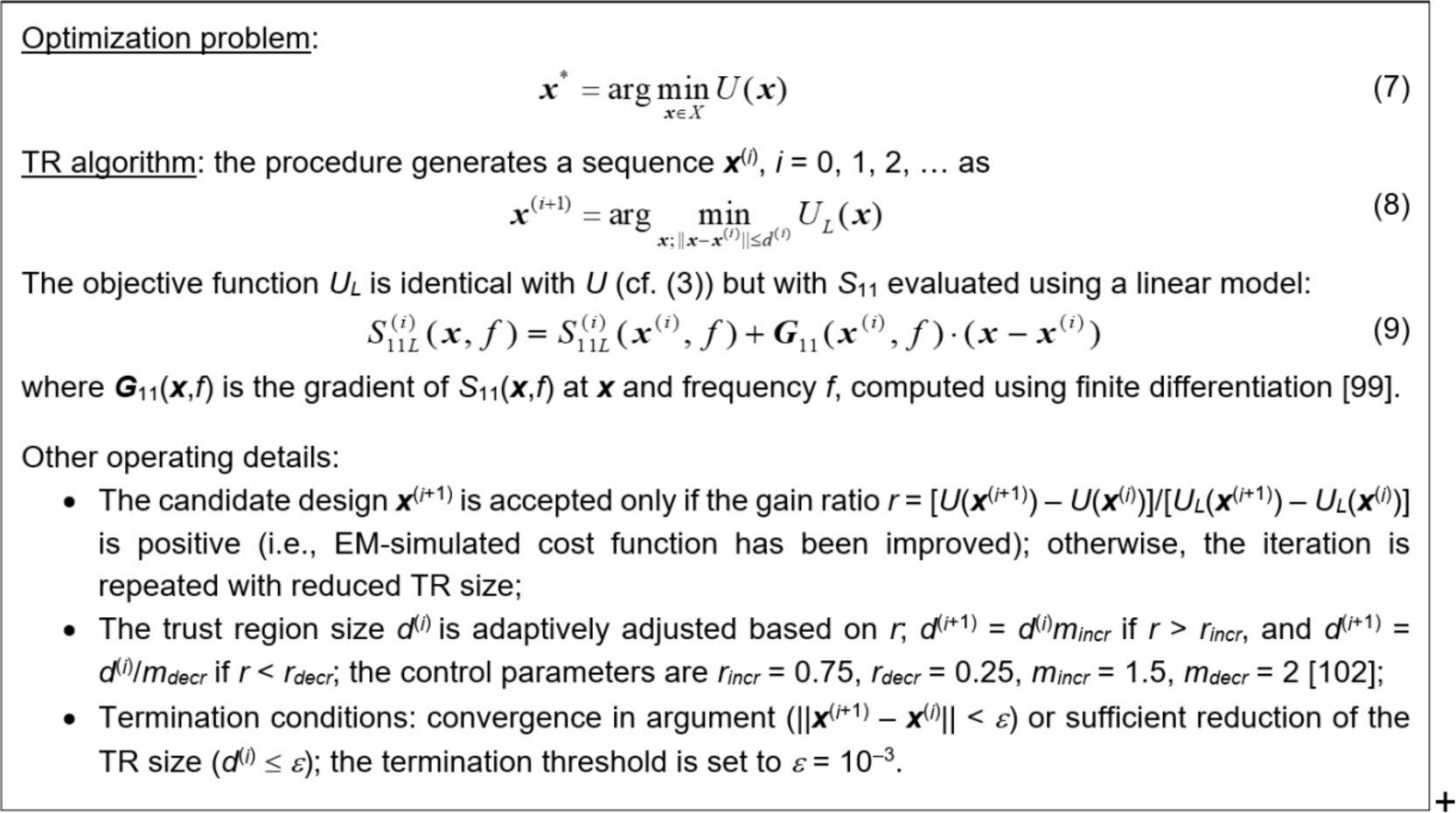
The outline of the TR algorithm.

The final design process stage is local tuning aimed at improving the antenna performance regarding the cost function *U* (here, in-band impedance matching enhancement). At this point, antenna topology is essentially fixed. Only the sizes/locations of its building blocks vary slightly. This research’s underlying algorithm is the trust-region (TR) routine with numerical gradients^98,99,102^, outline in Fig. 5. The optimization process is handled by solving a sequence of sub-problems (cf. (8)), each producing the next optimum approximation. The search is performed in the vicinity of the current design using a Taylor expansion model of frequency characteristics. The task (8) is resolved by means of the Sequential Quadratic Approximation (SQP) algorithm^100^ built in Matlab Optimization Toolbox^101^.

Each iteration costs *n* + 1 EM simulations, *n* being the number of decision variables. However, as some building blocks have minor effects on antenna responses (e.g., due to their specific allocation), the respective parameters are excluded from further optimization if their impact (detected in the first iteration) is low. This way, the cost-effectiveness of the refinement process is greatly improved. For additional cost reduction, the global design stage uses coarse-discretization EM analysis. It is replaced by a higher-fidelity one in the second stage (final tuning).

### Complete framework

**Fig. 6:**
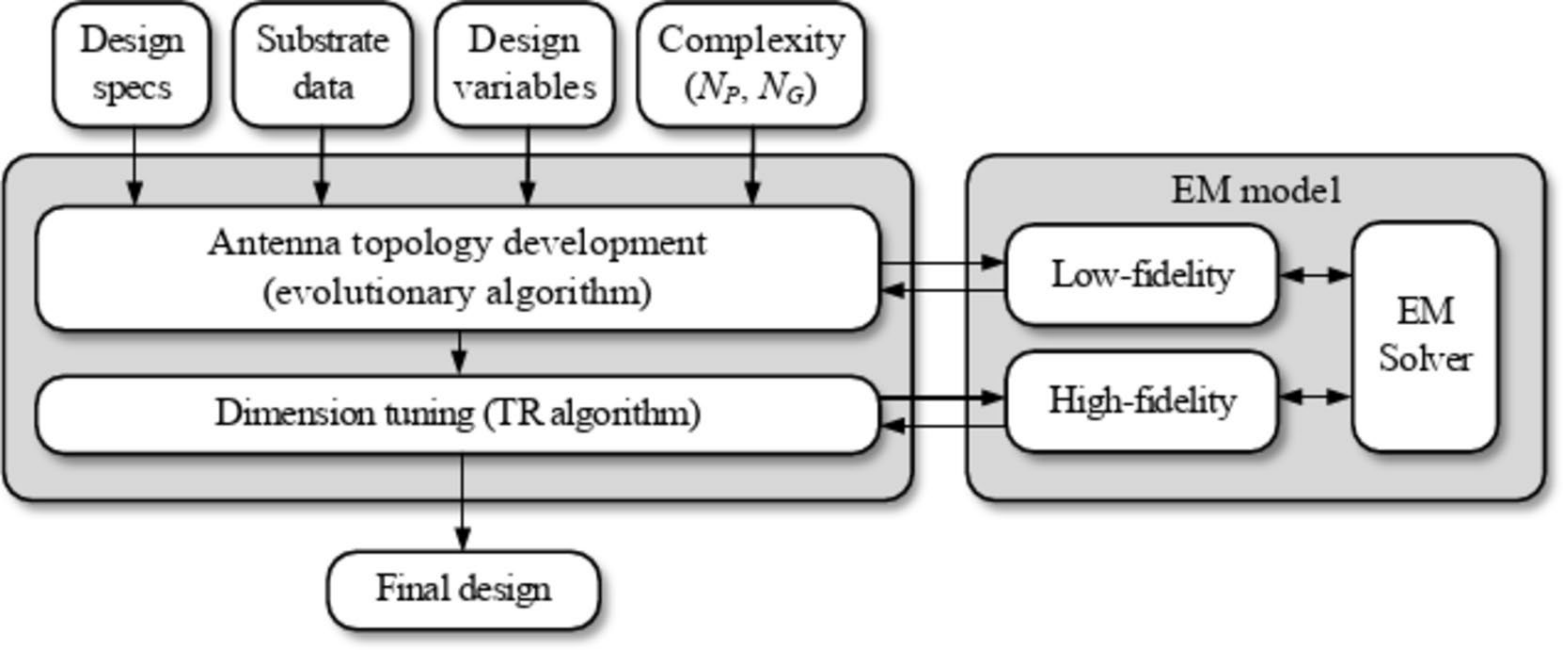
Unsupervised antenna design framework: the flow diagram.

The workflow of the suggested unsupervised design framework is illustrated in Fig. 6. As mentioned earlier, the antenna development process is specification-driven and requires no human expert input. The input parameters include parameters of the substrate (permittivity, thickness), substrate size (if treated as fixed), and target operating bands (cf. Section 2.3.1). The user may also decide the structure complexity by setting the number of active patches and gaps (*N*_*P*_ and *N*_*G*_, respectively). During the two design stages, the antenna topology is first decided upon through evolutionary optimization (global stage), and final tuning (local stage).

It should be reiterated that the proposed methodology does not require setting up any initial values of the design parameters. The first stage of the design process is executed using a global search routine, here, the evolutionary algorithm. Its initial population is established randomly. This is a considerable advantage of the proposed method over many other techniques, e.g., free-form topology optimization, which relies on local search procedures (typically gradient-based). Because the design variables are relative (only recomputed to absolute antenna dimensions concerning the assumed substrate size), the parameter bounds are set very wide (from almost zero to 0.9), eliminating the problem of meticulous bound adjustment.

##  Demonstration examples

This part of the paper demonstrates the capability of our procedure regarding the unsupervised antenna design. It is used to develop several broadband and multi-band structures operating at diverse frequency ranges. Identical algorithm setup is employed for all examples to indicate that no control parameter tuning is necessary. Experimental data for selected designs support numerical results. The rest of the section is arranged as detailed below. Section 3.1 discusses the design prerequisites. Section 3.2 through 3.10 provide the results for specific test cases, whereas Sect. 3.11 summarizes the findings.

### Experimental setup

The following arrangements have been made concerning the algorithm control parameters:

- Substrate: FR-4 (*ε*_*r*_ = 4.4, *h* = 1.0 mm);
- Model complexity: *N*_*P*_ = 5, *N*_*G*_ = 3;
- Fixed substrate size: *W* = 30 mm, *L* = 20 mm;
- Antenna topology development: control parameters as discussed in Sect. 2.3.2; computational budget 2000 EM simulations (100 iterations; population size *N* = 20);
- Local tuning: control parameters as discussed in Sect. 2.3.3.

Recall that topology development is carried out using a low-fidelity EM model (~ 60,000 mesh cells, typical evaluation time 20 s), whereas local tuning employs the high-fidelity model (~ 200,000 mesh cells, typical evaluation time one minute).

An identical setup is applied to all test cases of Sect. 3.2 through 3.10 to demonstrate that there is no need to adjust the control parameters to specific performance requirements (here, target operating bands). The only exception is a UWB antenna designed in Sect. 3.4, where the antenna size has been reduced to *W* = 25 mm, and *L* = 15 mm to show the capability of our framework to design more compact structures as well.

### Case I

**Fig. 7:**
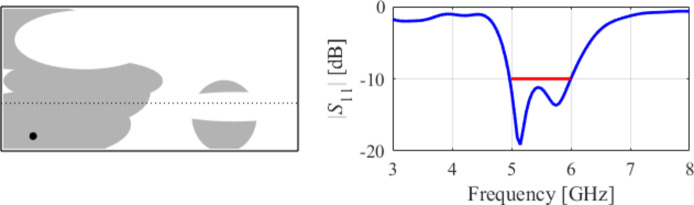
Case I: a single-band antenna working in the 5.0 GHz to 6.0 GHz range. Final geometry (ground plane marked using the dotted line) and the reflection response.

**Fig. 8:**
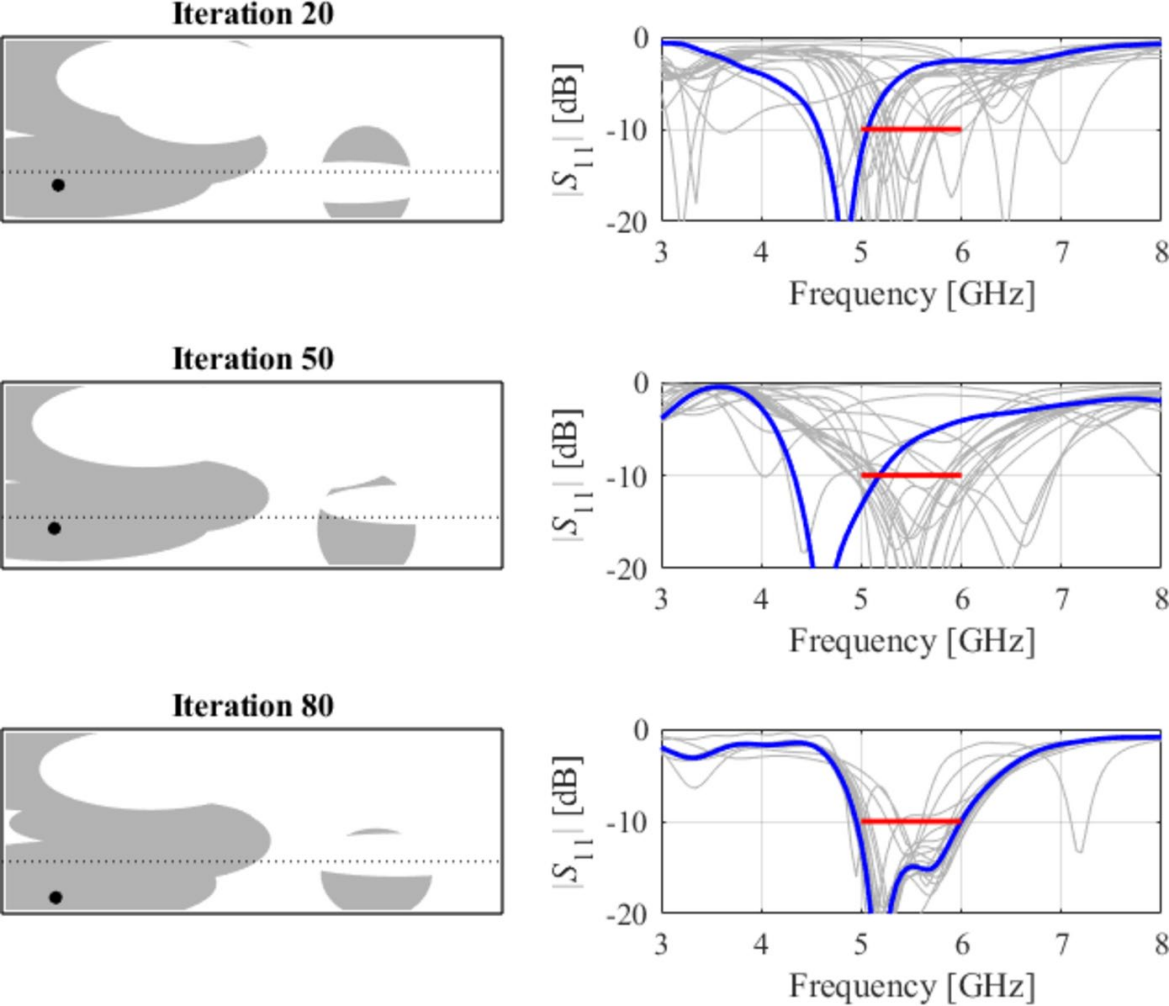
Case I: topology evolution at selected iterations of the global search procedure. The thick line corresponds to the best candidate architecture identified thus far. The gray lines mark the antenna characteristics at the current algorithm population.

**Fig. 9:**
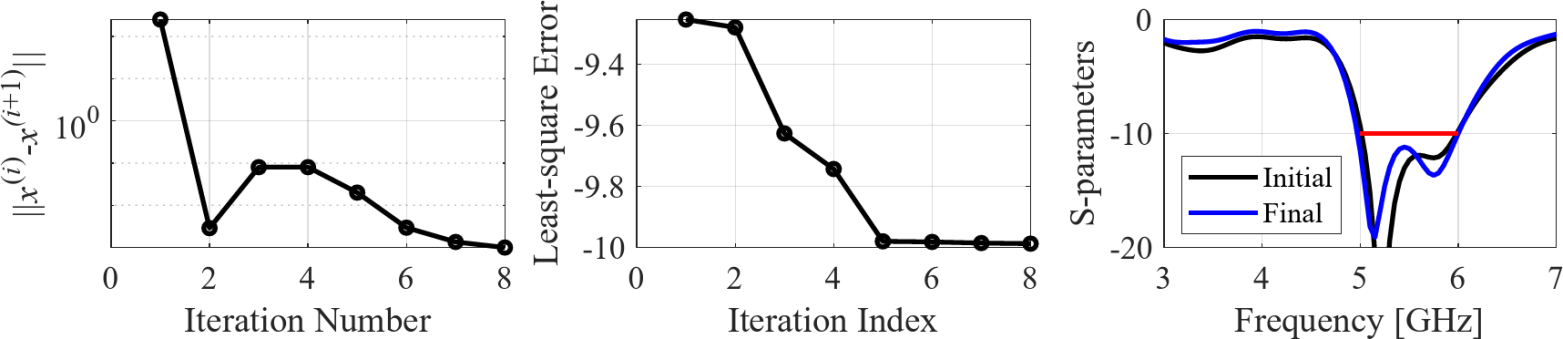
Case I: local parameter tuning using the TR algorithm: (**a**) convergence plot, (**b**) objective function evolution, (**c**) initial and final |*S*_11_| versus frequency.

The initial test case is a single-band antenna to operate in the 5.0 GHz to 6.0 GHz band. Figure 7 illustrates the final antenna geometry obtained with our technique and the reflection response. For an additional illustration, Fig. 8 shows several snapshots from the first design stage (topology evolution). Figure 9 shows the convergence plot for local tuning, a history of the minimax objective function, and a comparison of the initial (after a global search) and final |*S*_11_| characteristics. It can be noted that the global search stage already produces a good-quality antenna, whereas local tuning improves impedance matching by almost 1 dB within several iterations. Design specifications are fulfilled for the final structure. One should reiterate that the design process is entirely specification-driven, and no human expert is involved.

### Case II

**Fig. 10:**
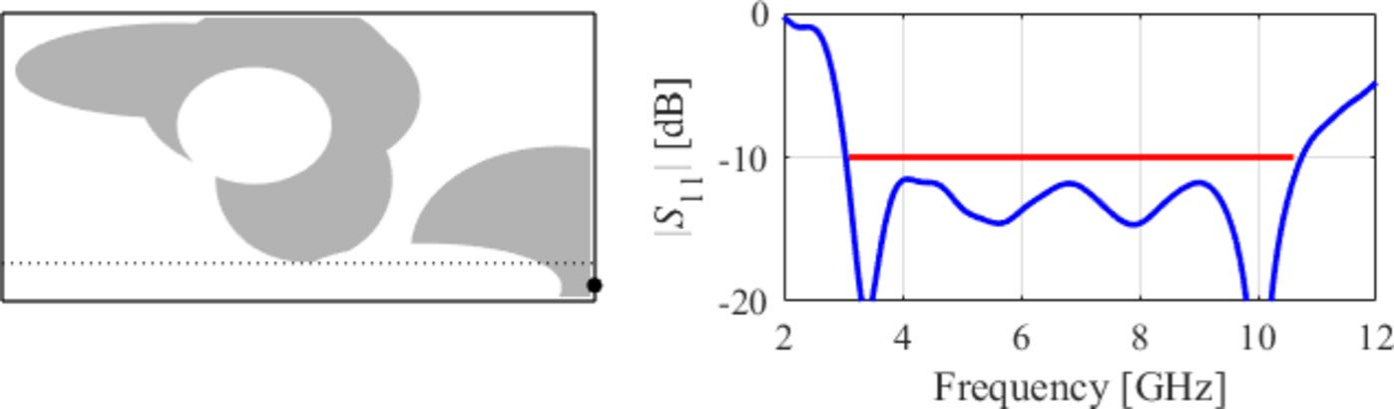
Case II: a UWB antenna working in the 3.1 GHz to 10.6 GHz range. Final geometry (ground plane marked using the dotted line) and the reflection response.

**Fig. 11:**
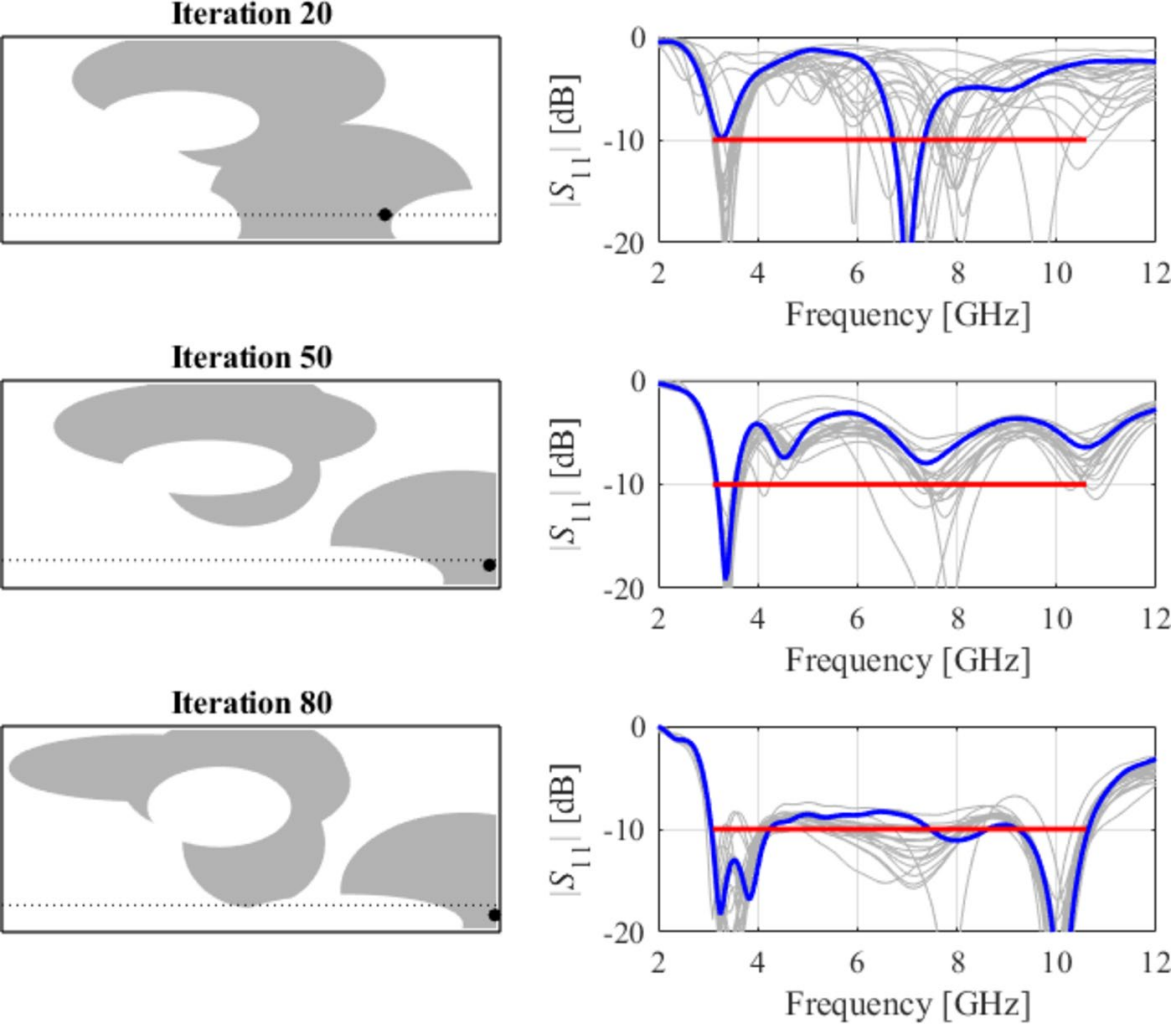
Case II: topology evolution at selected iterations of the global search procedure. The thick line corresponds to the best candidate architecture identified thus far. The gray lines mark the antenna characteristics at the current algorithm population.

**Fig. 12:**
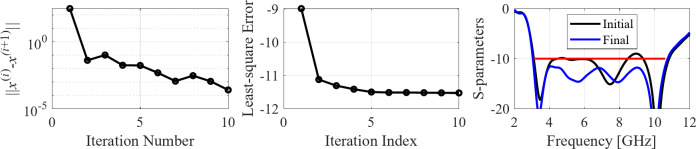
Case II: local parameter tuning using the TR algorithm: (a) convergence plot, (b) objective function evolution, (c) initial and final |*S*_11_| versus frequency.

The second example is an ultra-wideband (UWB) structure working in the 3.1 GHz to 10.6 GHz band. The final geometry produced by the proposed framework and the corresponding reflection response are shown in Fig. 10. Figures 11 and 12 illustrate the topology evolution and the details of local tuning.

**Fig. 13:**
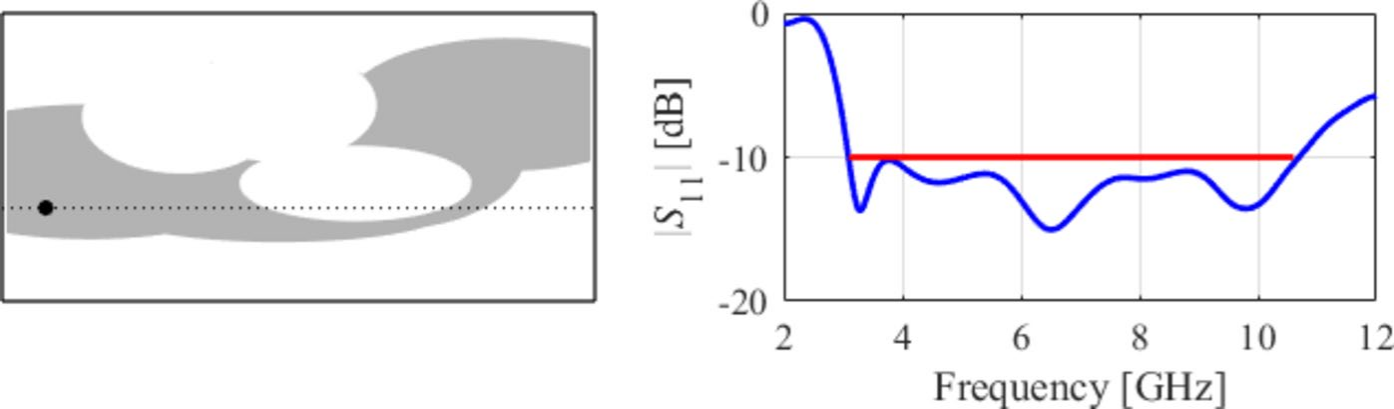
Case III: a compact UWB antenna working in the 3.1 GHz to 10.6 GHz range (size 15 × 25 mm). Final geometry (ground plane marked using the dotted line) and the reflection response.

**Fig. 14:**
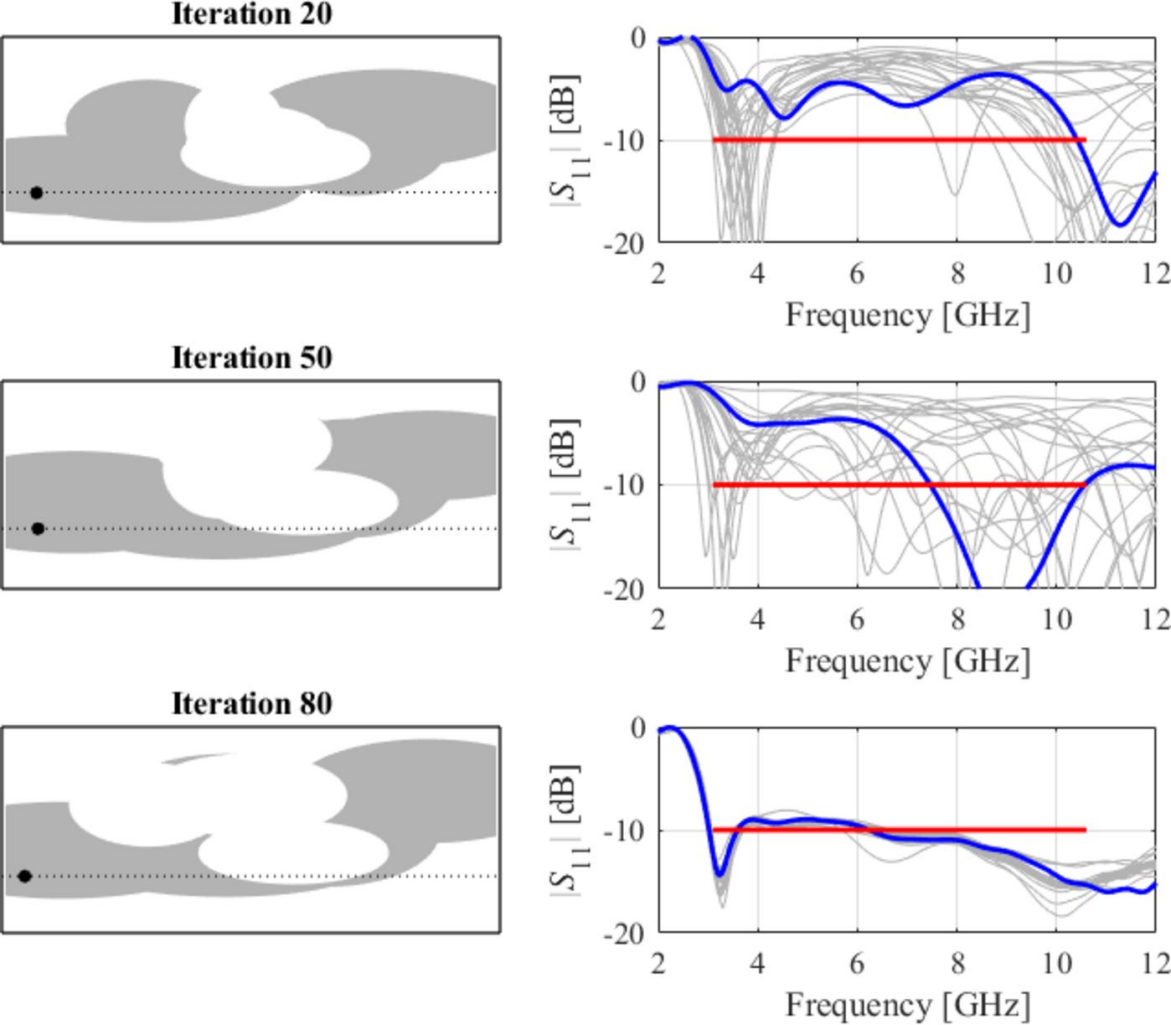
Case III: topology evolution at selected iterations of the global search procedure. The thick line corresponds to the best candidate architecture identified thus far. The gray lines mark the antenna characteristics at the current algorithm population.

**Fig. 15:**
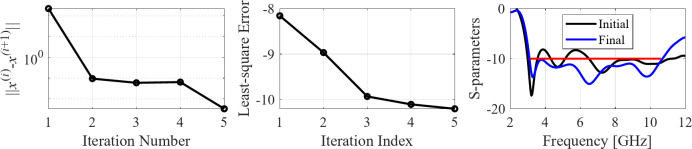
Case III: local parameter tuning using the TR algorithm: (a) convergence plot, (b) objective function evolution, (c) initial and final |*S*_11_| versus frequency.

### Case III

The third test scenario is also a UWB antenna. However, this antenna is implemented on a smaller substrate (15 mm × 20 mm in contrast to 20 mm × 30 mm for Case II). Nonetheless, design specifications have also been fulfilled in this case, as shown in Fig. 13. The snapshots of global search and local parameter adjustment can be found in Figs. 14 and 15, respectively.

### Case IV

**Fig. 16:**
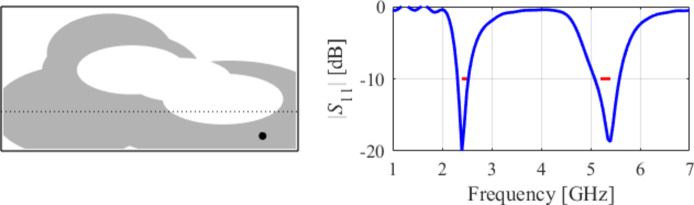
Case IV: a dual-band antenna working in the 2.4 GHz to 2.5 GHz and 5.2 GHz to 5.4 GHz range. Final geometry (ground plane marked using the dotted line) and the reflection response.

**Fig. 17:**
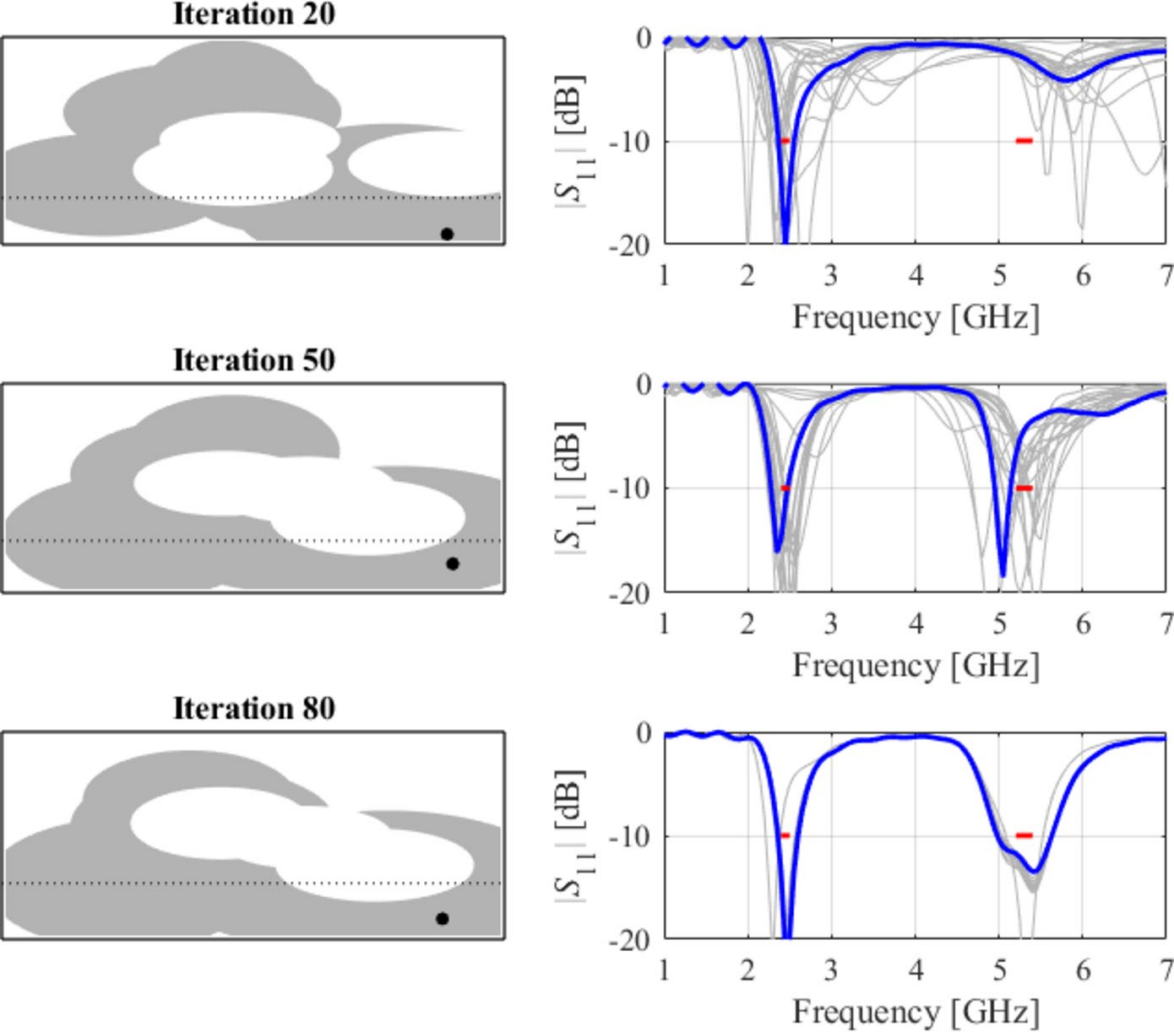
Case IV: topology evolution at selected iterations of the global search procedure. The thick line corresponds to the best candidate architecture identified thus far. The gray lines mark the antenna characteristics at the current algorithm population.

**Fig. 18:**
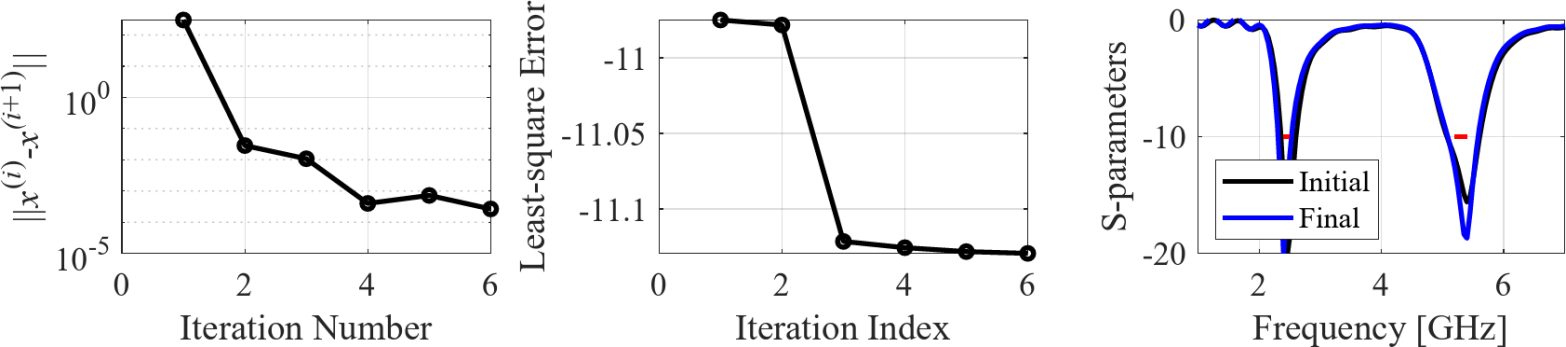
Case IV: local parameter tuning using the TR algorithm: (a) convergence plot, (b) objective function evolution, (c) initial and final |*S*_11_| versus frequency.

The next case involves a dual-band antenna operating from 2.4 GHz to 2.5 GHz and 5.2 GHz to 5.4 GHz. It can be noted that the design specifications are fulfilled with a good margin (cf. Fig. 16), and the global search already yields a solution of a good quality, requiring only a slight improvement through local tuning, see Figs. 17 and 18.

### Case V

**Fig. 19:**
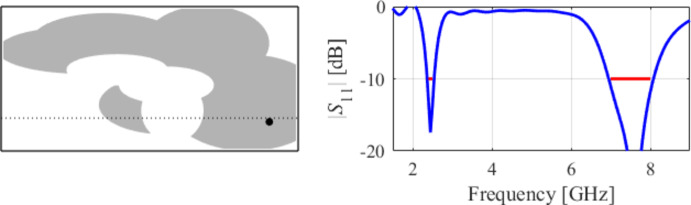
Case V: a dual-band antenna working in the 2.4 GHz to 2.5 GHz and 7.0 GHz to 8.0 GHz range. Final geometry (ground plane marked using the dotted line) and the reflection response.

**Fig. 20:**
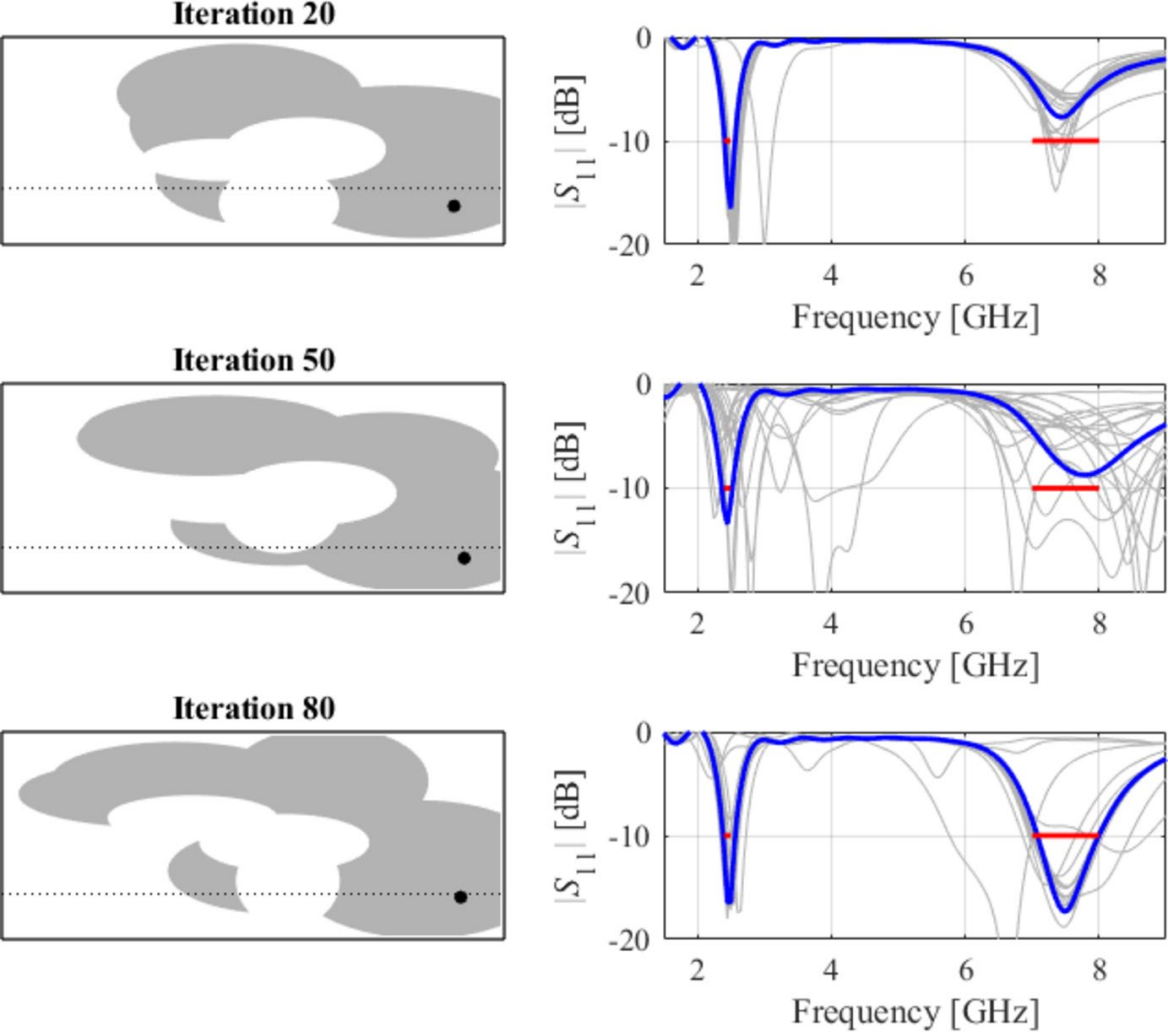
Case V: topology evolution at selected iterations of the global search procedure. The thick line corresponds to the best candidate architecture identified thus far. The gray lines mark the antenna characteristics at the current algorithm population.

**Fig. 21:**
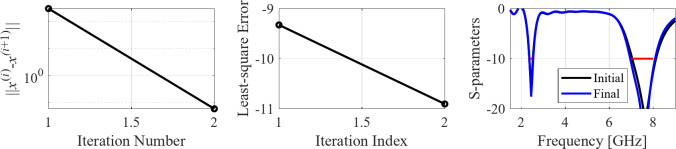
Case V: local parameter tuning using the TR algorithm: (a) convergence plot, (b) objective function evolution, (c) initial and final |*S*_11_| versus frequency.

The following test scenario is a dual-band antenna operating from 2.4 GHz to 2.5 GHz and 7.0 GHz to 8.0 GHz. Also, in this case, the global search stage yields a good-quality design, so the local tuning only leads to minor dimension changes. The final geometry is shown in Fig. 19, whereas Figs. 20 and 21 illustrate antenna topology evolution and gradient-based tuning, accordingly.

### Case VI

**Fig. 22:**
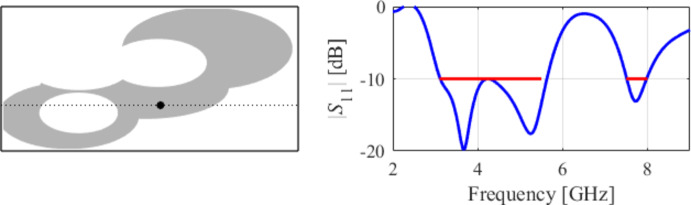
Case VI: a dual-band antenna working in the 3.1 GHz to 5.5 GHz and 7.5 GHz to 8.0 GHz range. Final geometry (ground plane marked using the dotted line) and the reflection response.

**Fig. 23:**
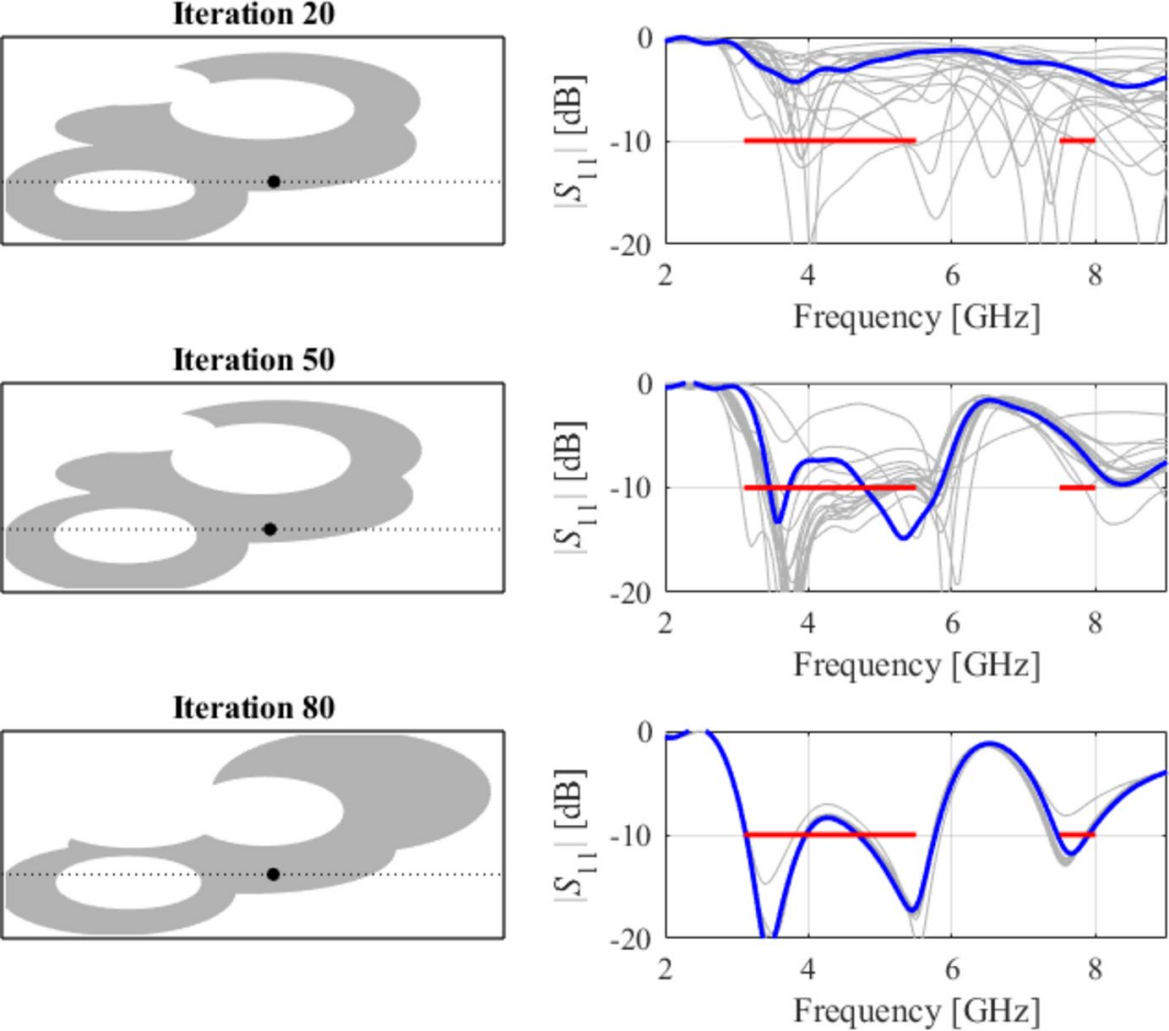
Case VI: topology evolution at selected iterations of the global search procedure. The thick line corresponds to the best candidate architecture identified thus far. The gray lines mark the antenna characteristics at the current algorithm population.

**Fig. 24:**
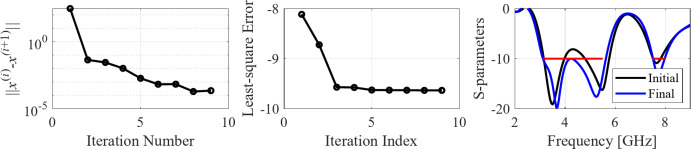
Case VI: local parameter tuning using the TR algorithm: (a) convergence plot, (b) objective function evolution, (c) initial and final |*S*_11_| versus frequency.

The next example is a broadband dual-band antenna operating from 3.1 GHz to 5.5 GHz and 7.5 GHz to 8.0 GHz. As indicated in Fig. 22, the final design fulfills the specifications, although it is a considerably more complex scenario due to broadband requirements in the lower band. Here, the global search results (Fig. 23) need to be adjusted (cf. Fig. 24) to meet the requirements eventually.

### Case VII

**Fig. 25:**
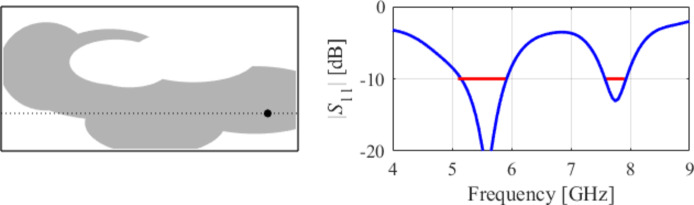
Case VII: a dual-band antenna working in the 5.1 GHz to 5.9 GHz and 7.6 GHz to 7.9 GHz range. Final geometry (ground plane marked using the dotted line) and the reflection response.

**Fig. 26:**
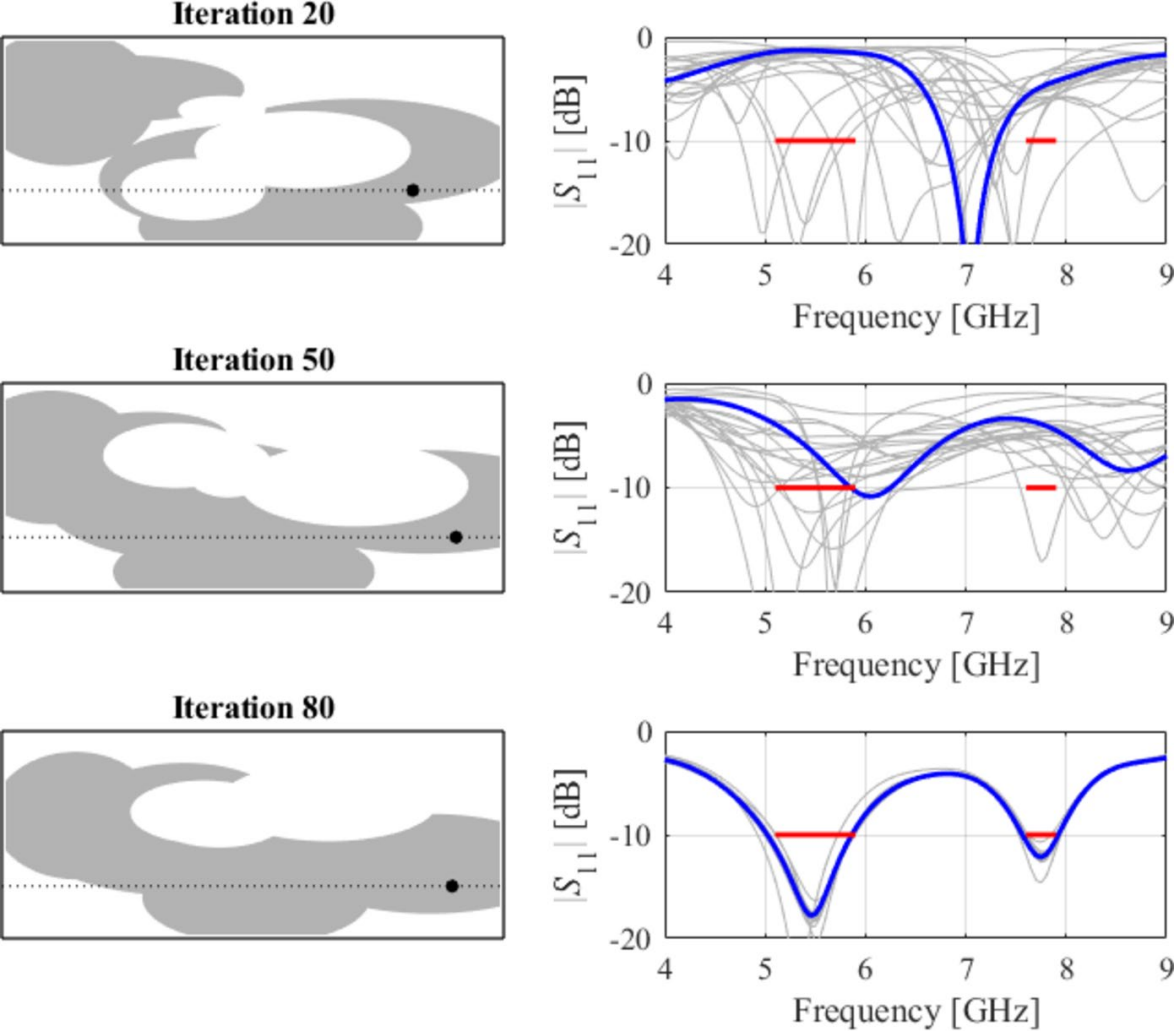
Case VII: topology evolution at selected iterations of the global search procedure. The thick line corresponds to the best candidate architecture identified thus far. The gray lines mark the antenna characteristics at the current algorithm population.

**Fig. 27:**
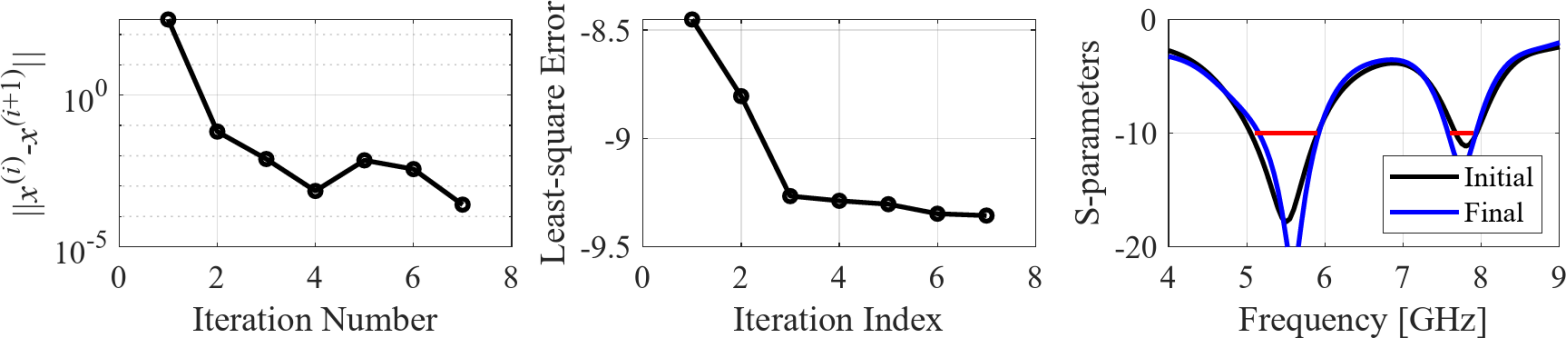
Case VII: local parameter tuning using the TR algorithm: (a) convergence plot, (b) objective function evolution, (c) initial and final |*S*_11_| versus frequency.

The seventh test case is a dual-band antenna operating from 5.1 GHz to 5.9 GHz and 7.6 GHz to 7.9 GHz. The design produced by our technique almost fulfills the specifications (maximum in-band |*S*_11_| of around − 9.5 dB), cf. Figure 25. Figures 26 and 27 illustrate the global and local search stages. The latter improves the design by around 1 dB within a few iterations.

### Case VIII

**Fig. 28:**
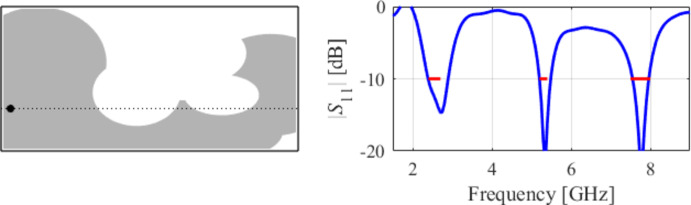
Case VIII: a triple-band antenna working in the 2.4 GHz to 2.5 GHz, 5.2 GHz to 5.4 GHz, and 7.5 GHz to 8.0 GHz range. Final geometry (ground plane marked using the dotted line) and the reflection response.

**Fig. 29:**
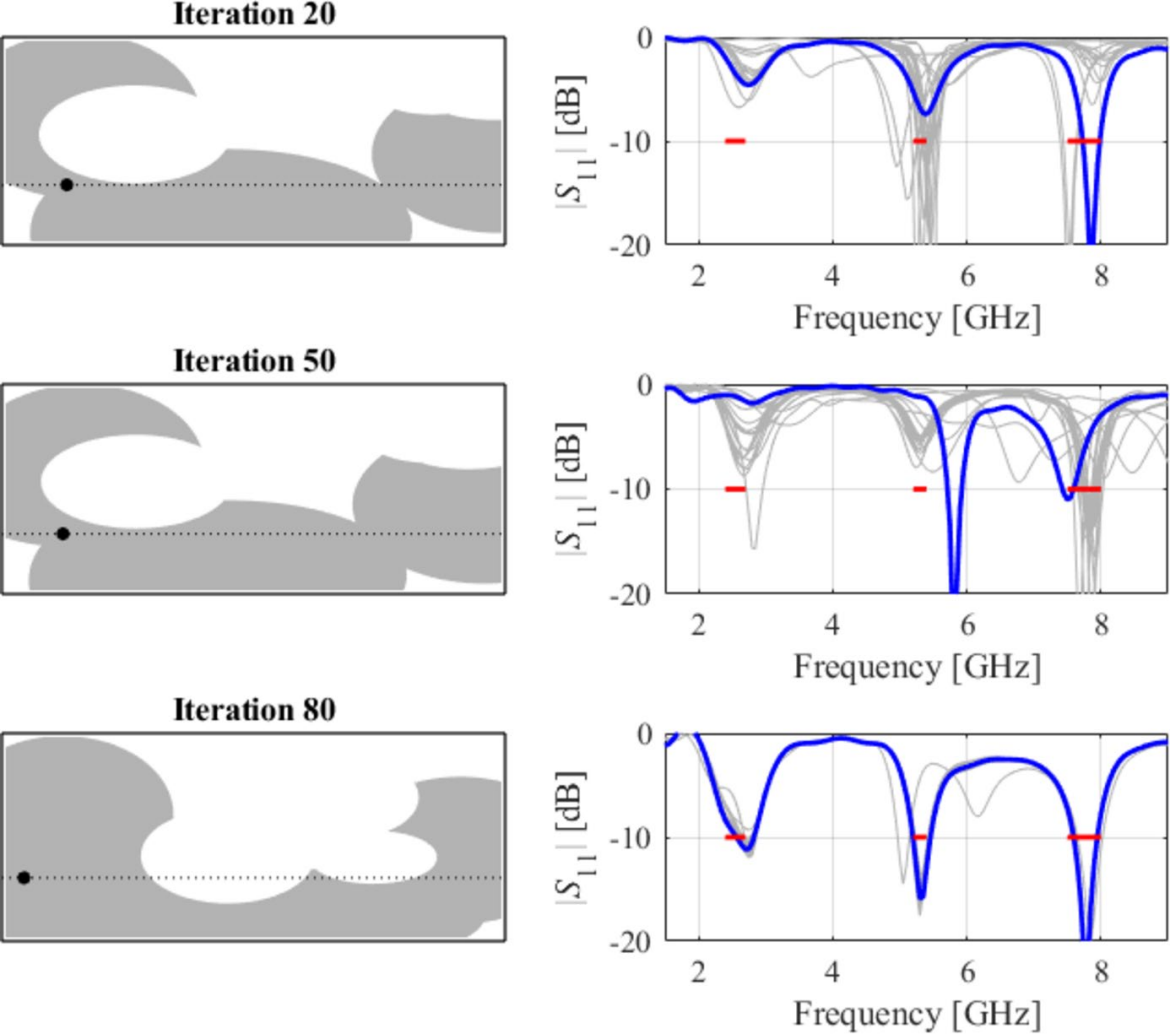
Case VIII: topology evolution at selected iterations of the global search procedure. The thick line corresponds to the best candidate architecture identified thus far. The gray lines mark the antenna characteristics at the current algorithm population.

**Fig. 30:**
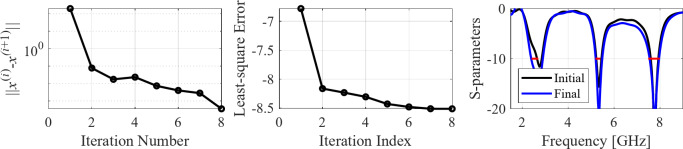
Case VIII: local parameter tuning using the TR algorithm: (a) convergence plot, (b) objective function evolution, (c) initial and final |*S*_11_| versus frequency.

The eighth example involves a triple-band antenna operating in the ranges 2.4 GHz to 2.5, GHz, 5.2 GHz to 5.4 GHz, and 7.5 GHz to 8.0 GHz. As indicated in Figs. 28 and 29, and 30, for this case, the specifications are met in the first two operating bands and slightly violated in the upper band (7.5 GHz to 8.0 GHz). Notwithstanding, the antenna has been generated using the same algorithmic setup as for the previous test cases, and a triple-band operation was achieved using low-complexity architecture.

### Case IX

The final example is again a triple-band antenna. The target operating frequency bands are 3.6 GHz to 3.7 GHz, 5.4 GHz to 5.5 GHz, and 9.8 GHz to 10.2 GHz.

**Fig. 31:**
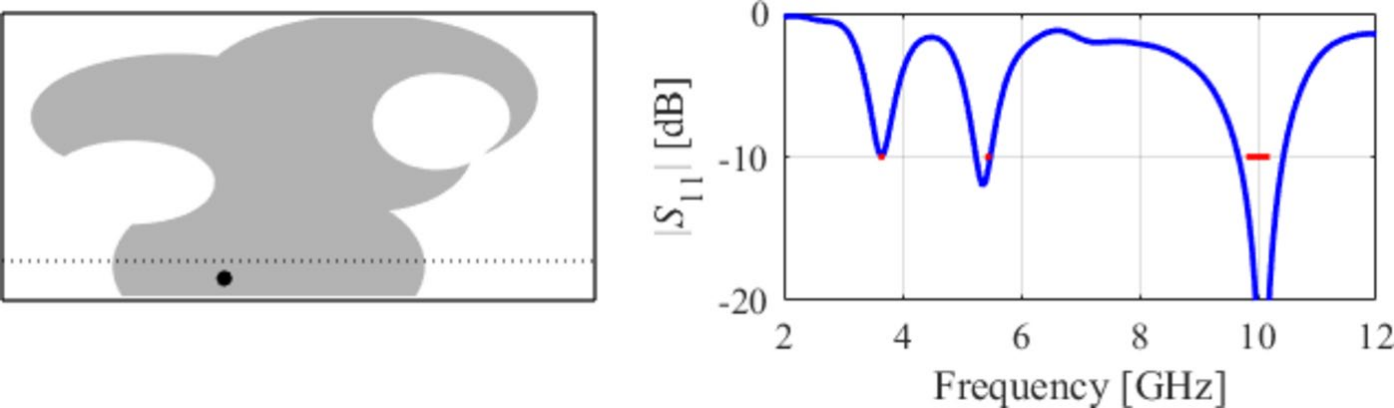
Case IX: a triple-band antenna working in the 3.6 GHz to 3.7 GHz, 5.4 GHz to 5.5 GHz, and 9.8 GHz to 10.2 GHz range. Final geometry (ground plane marked using the dotted line) and the reflection response.

**Fig. 32:**
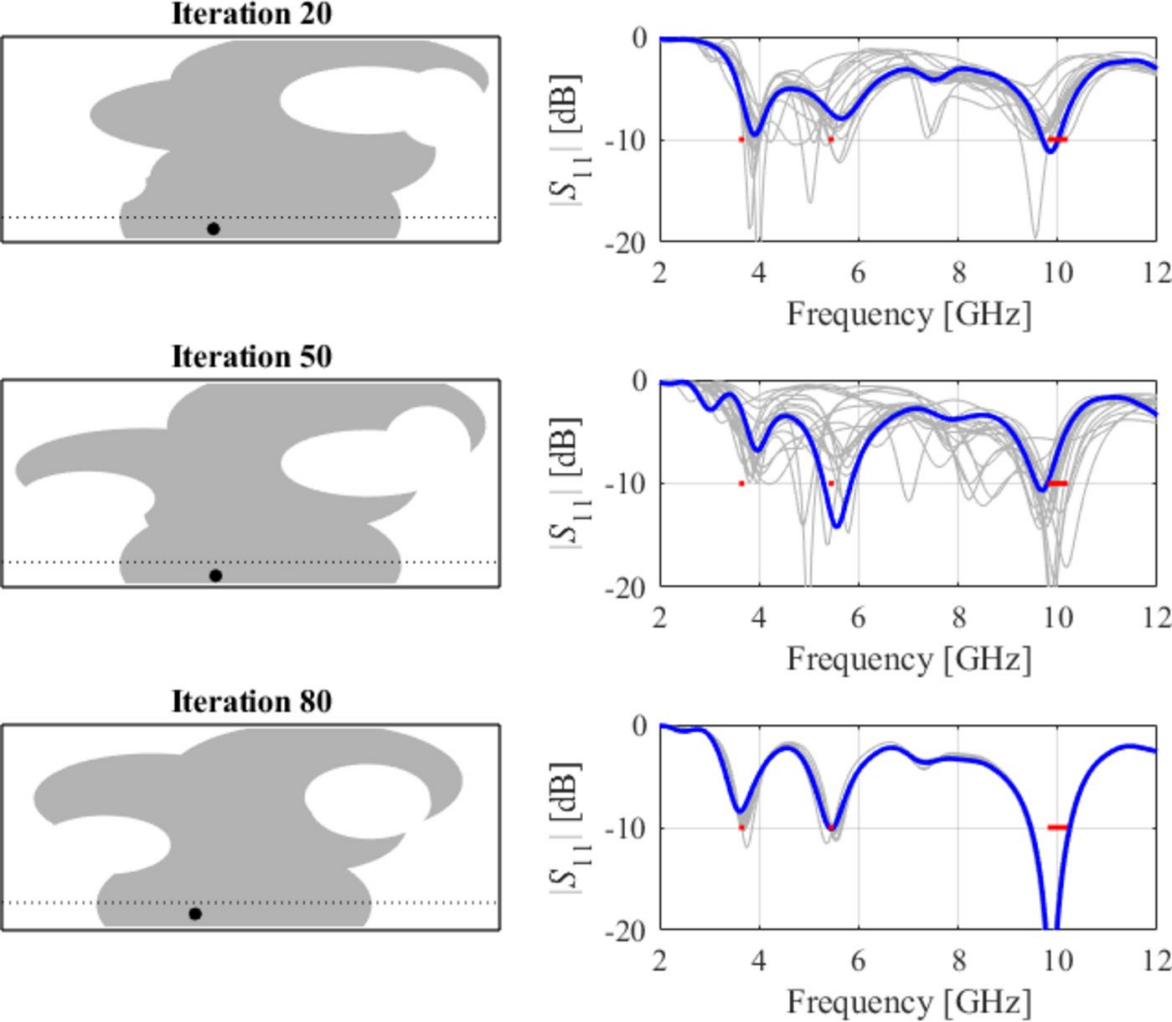
Case IX: topology evolution at selected iterations of the global search procedure. The thick line corresponds to the best candidate architecture identified thus far. The gray lines mark the antenna characteristics at the current algorithm population.

**Fig. 33:**
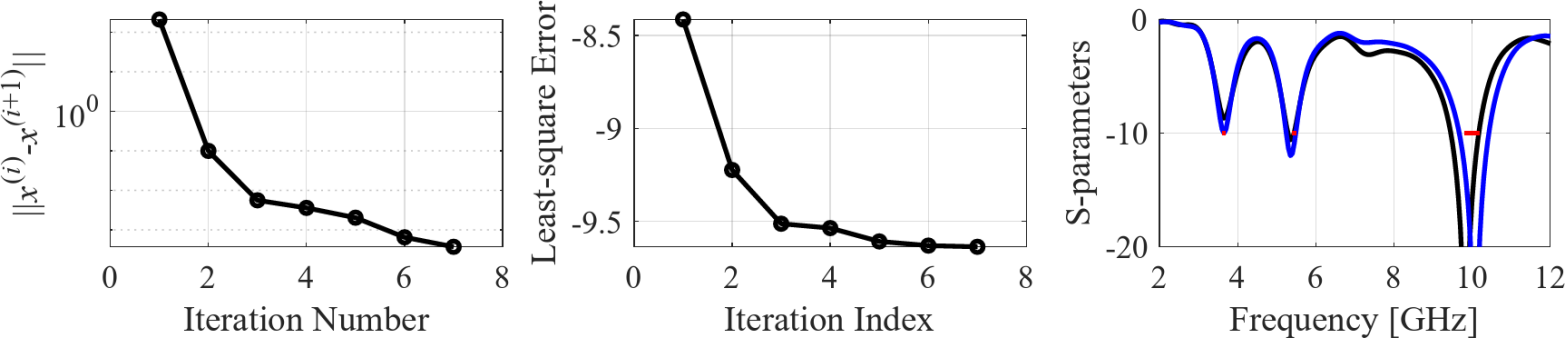
Case IX: local parameter tuning using the TR algorithm: (a) convergence plot, (b) objective function evolution, (c) initial and final |*S*_11_| versus frequency.

As indicated in Figs. 31 and 32, and 33, for this case, the specifications are met in the first two operating bands and slightly violated in the upper band (7.5 GHz to 8.0 GHz). Notwithstanding, the antenna has been generated using the same algorithmic setup as for the previous test cases, and a triple-band operation was achieved using low-complexity architecture.

**Fig. 34:**
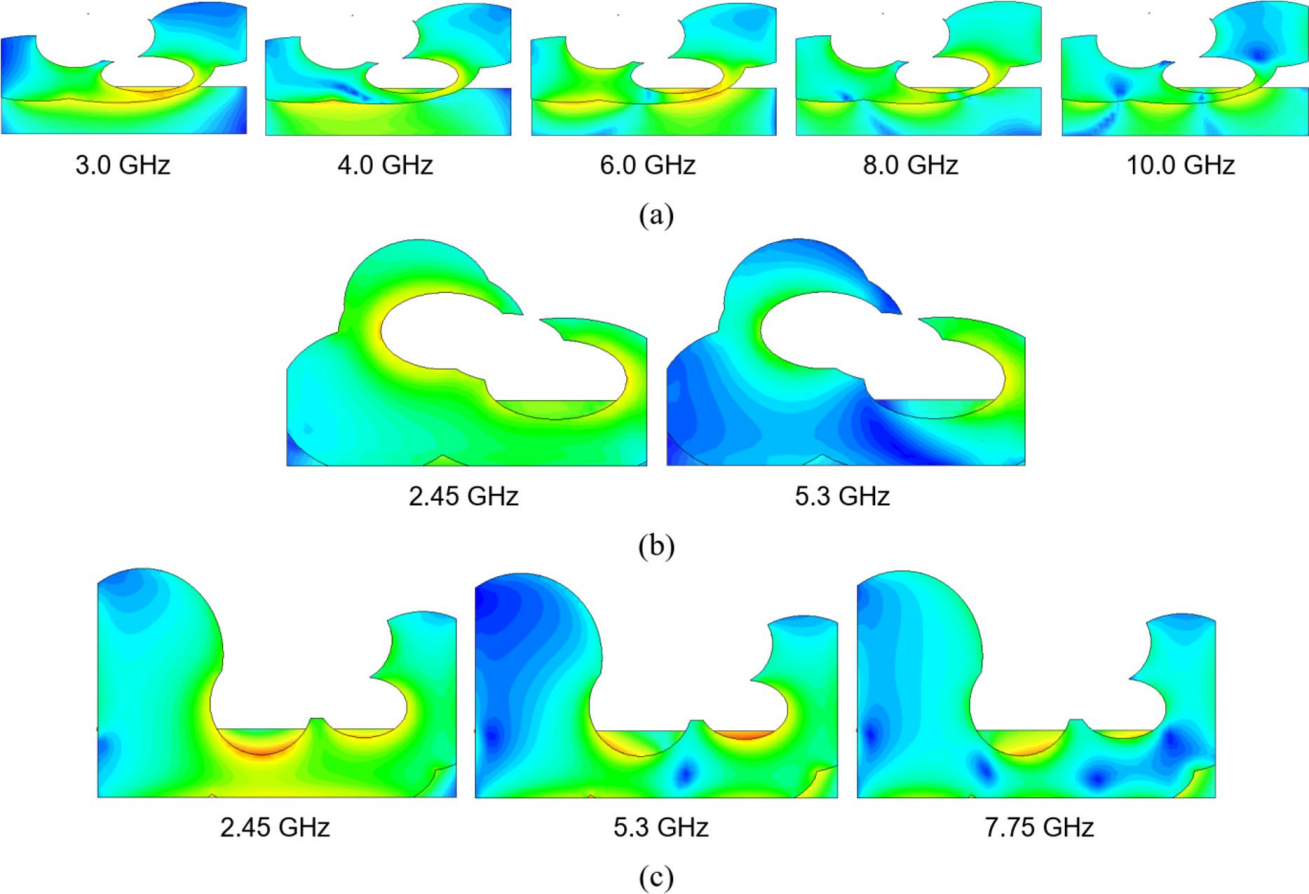
Surface current distributions for selected designs: (a) Case III, (b) Case IV, (c) Case VIII. The pictures underscore the utilization of the various parts of the antenna geometry at various frequencies. This corroborates the relevance of allocating the antenna’s building blocks during the topology evolution process.

### Summary of findings

The results encapsulated in Sect. 3.2 through 3.10 conclusively demonstrate the efficacy and versatility of the proposed unsupervised antenna design strategy. Using a few elementary building blocks in conjunction with computational intelligence and rigorous optimization methods enables the automated design of a large variety of structures. These include single-band, dual-band, and triple-band devices, broadband antennas, and UWB radiators.

Figure 34 shows surface current distributions at various frequencies, scattered across the UWB band for Case III, and allocated at the centers of the respective operating bands for Cases IV and VIII. These pictures indicate the utilization of the various parts of the antenna geometry at different parts of the spectrum, demonstrating how particular building blocks have been allocated in the topology evolution process to ensure adequate antenna operation at the mentioned frequencies.

The design process of these diverse structures is purely specification-driven, and it does not require any adjustment of the algorithm setup. One of the critical components of the presented methodology is a combination of flexible parameterization, global search (for antenna architecture evolution), and local tuning for improving antenna performance, here, impedance matching. Furthermore, the antenna development process is realized using moderate expenses, amounting to 2000 low-fidelity EM analyses and between 100 and 300 high-fidelity EM simulations. This corresponds to the total CPU time of only around fifteen hours, which is very practical.

As elucidated earlier, the antenna performance is judged based on the value of the objective function, which, in this work, is a minimax function established for the reflection response. Consequently, the algorithm (its global and local parts) works towards improving *U*(***x***). If the value of the objective function falls below − 10 dB, the maximum in-band reflection level does not exceed the acceptance threshold of − 10 dB. Due to the stochastic nature of the evolutionary algorithm and ample parameter space (a few dozen decision variables), the procedure generally yields a different design each time it is executed. Consequently, there is no guarantee that any particular outcome is globally optimal. At this point, it should be emphasized that all antenna designs presented in the literature are always sub-optimal, not only due to the lack of appropriate optimization but—most importantly—due to considering a dramatically restricted number of topological options (often just one). The advantage of the presented approach is that it allows for considering a much more comprehensive range of antenna architectures while simultaneously optimizing the specific geometry dimensions.

##  Experimental validation

**Fig. 35:**
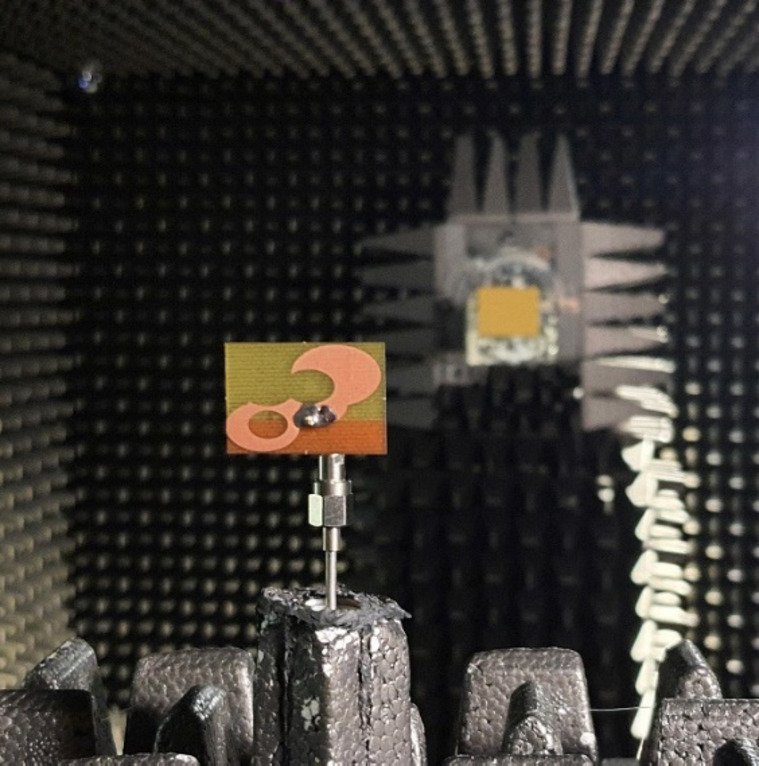
Experimental setup in the anechoic chamber (Case VI).

**Fig. 36:**
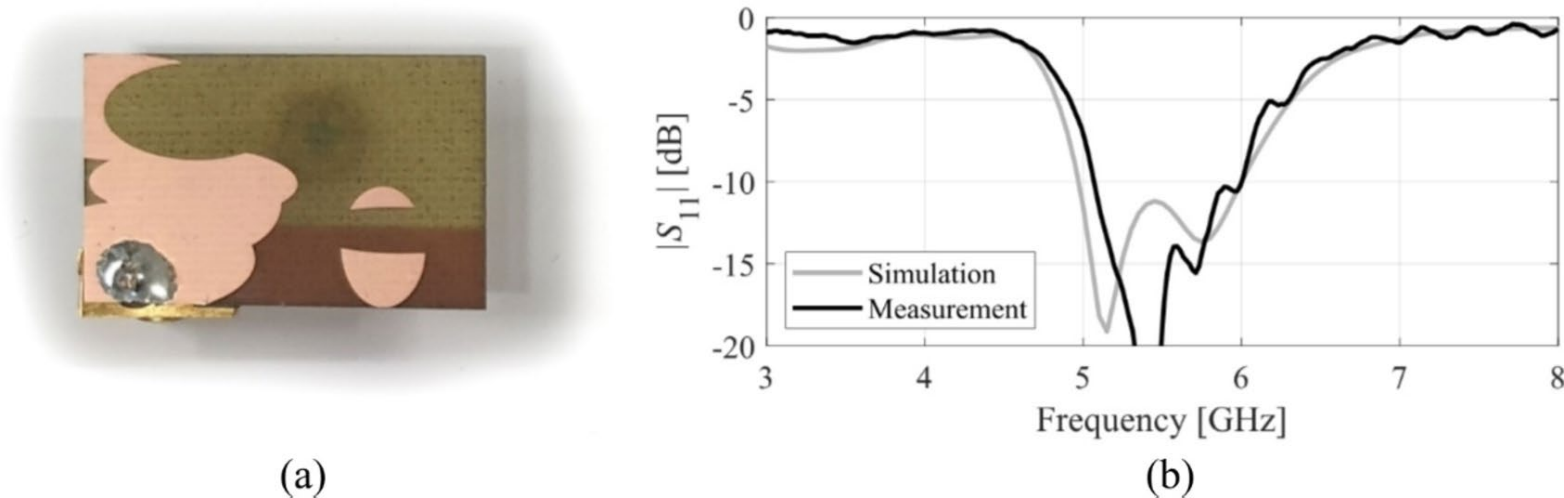
Case I: (**a**) antenna prototype (cf. Fig. 10), (**b**) EM-simulated and measured |*S*_11_|.

**Fig. 37:**
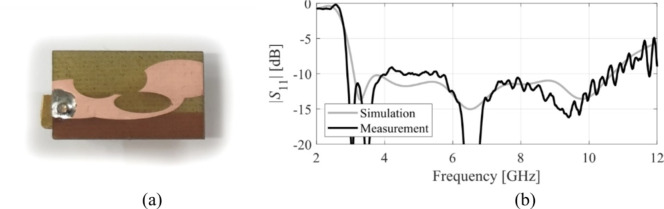
Case III: (**a**) antenna prototype (cf. Fig. 13), (**b**) EM-simulated and measured |*S*_11_|.

**Fig. 38:**
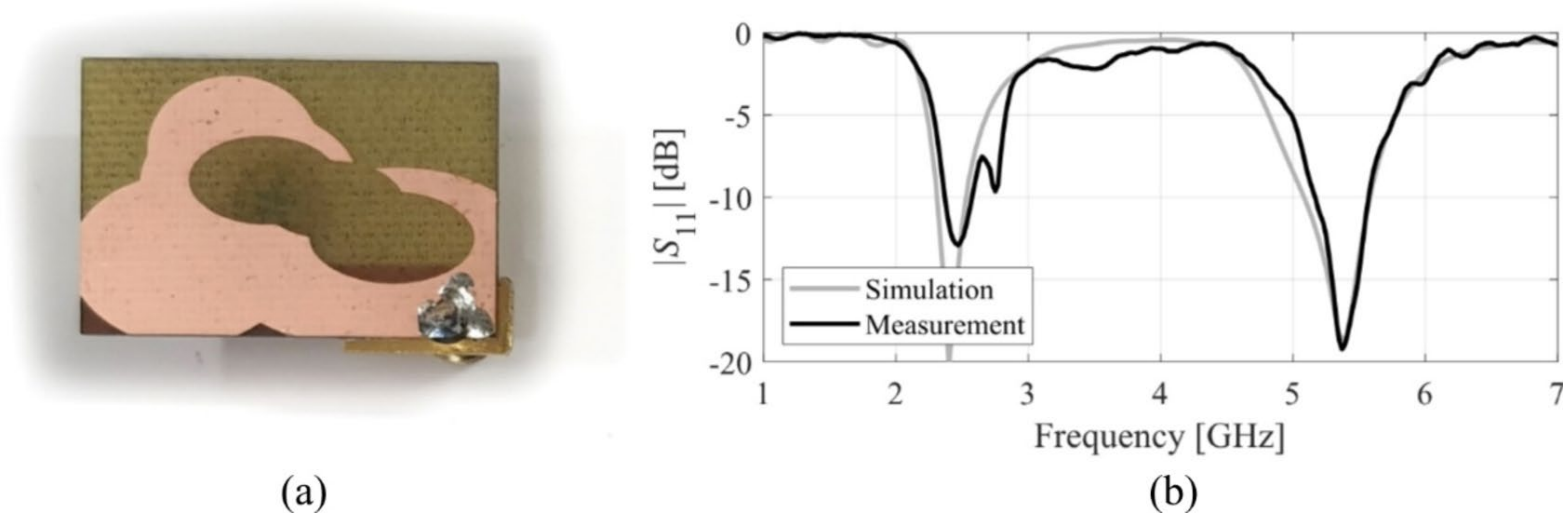
Case IV: (**a**) antenna prototype (cf. Fig. 16), (**b**) EM-simulated and measured |*S*_11_|.

**Fig. 39:**
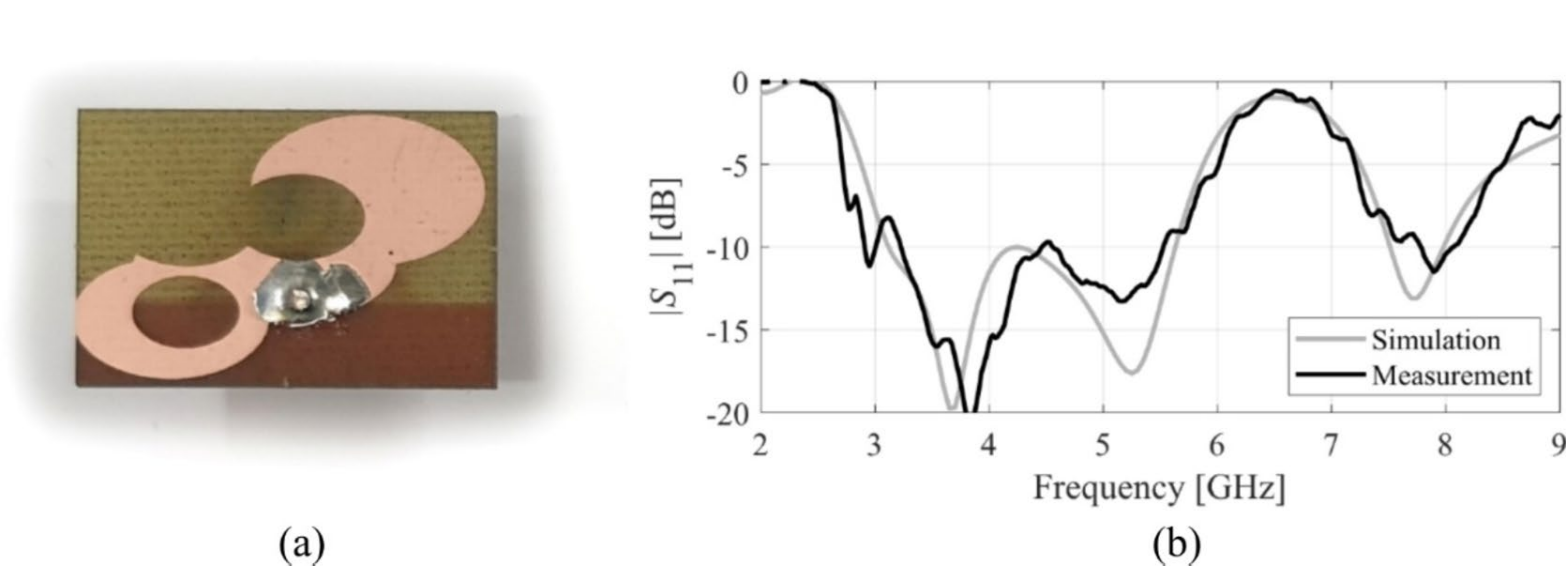
Case VI: (**a**) antenna prototype (cf. Fig. 22), (**b**) EM-simulated and measured |*S*_11_|.

**Fig. 40:**
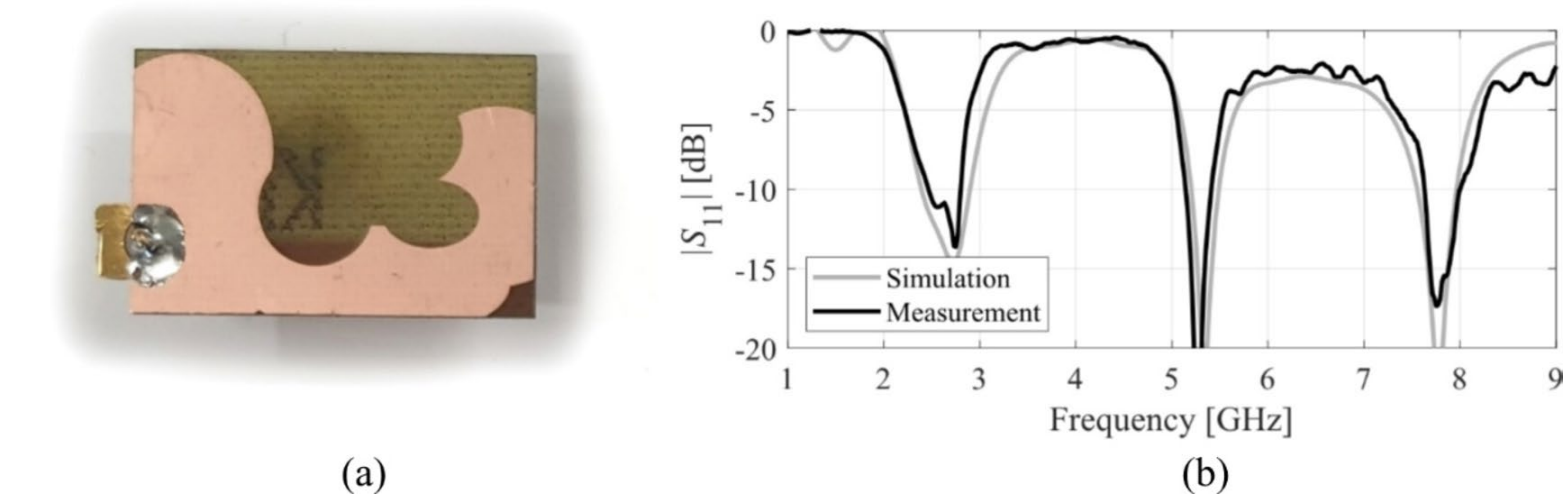
Case VII: (**a**) antenna prototype (cf. Fig. 28), (**b**) EM-simulated and measured |*S*_11_|.

**Fig. 41:**
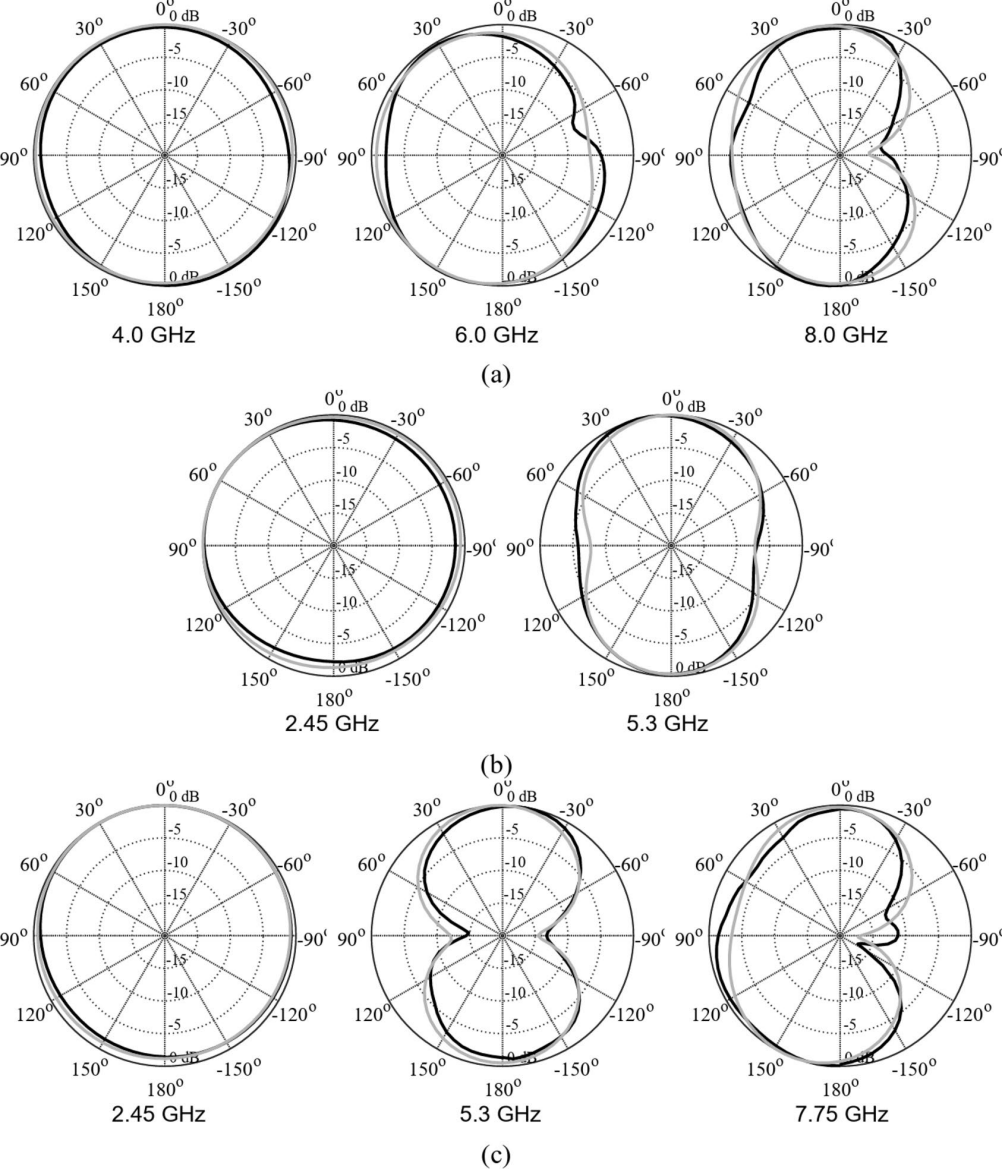
H-plane patterns for selected test cases: (a) Case III, (b) Case IV, (c) Case VIII. EM simulations and measurements shown using gray and black lines, respectively.

**Fig. 42:**
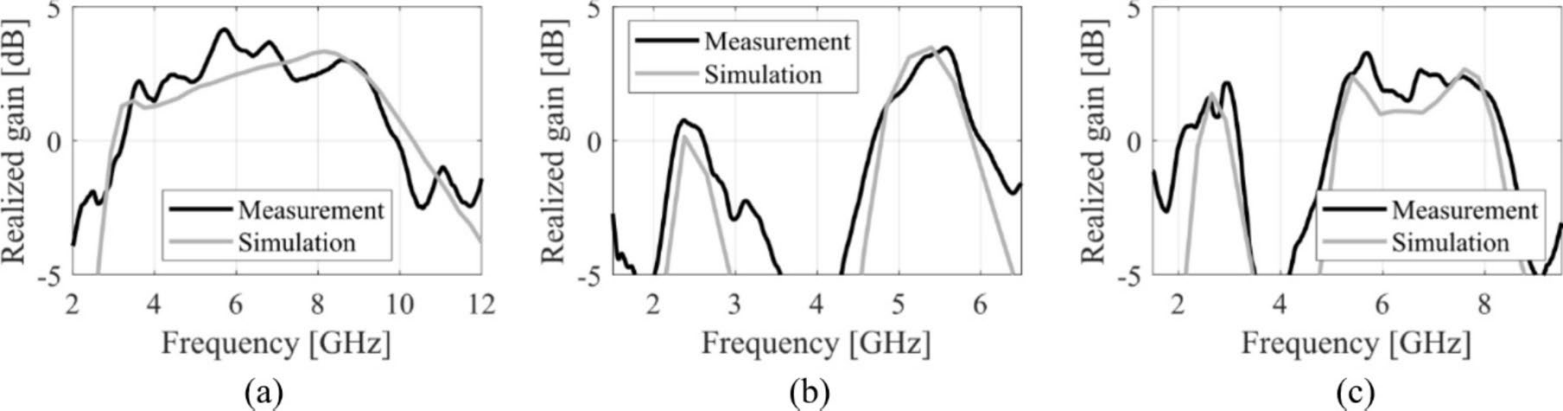
Realized gain for selected designs: (**a**) Case III, (**b**) Case IV, (**c**) Case VIII. EM simulations and measurements shown using gray and black lines, respectively.

The designs produced in Sect. 3 have been prototyped and experimentally validated. For the sake of brevity, the experimental data for five selected designs is included here, specifically Cases I, III, IV, VI, and VIII. Figure 35 illustrates the measurement setup in the anechoic chamber for Case VI (the same setup was used for all antennas). Figures 36, 37, 38 and 39, and 40 show the photographs of antenna prototypes for the respective cases and the comparison between EM-evaluated and measured |*S*_11_| characteristics. As observed, these are well-aligned. Minor discrepancies stem from fabrication and assembly imperfections and the effects of the SMA connector not included in the EM model. Figures 41 and 42 show EM-evaluated and measured radiation patterns and realized gain for selected designs, specifically, Cases III, IV, and VIII.

##  Conclusion

This research proposed an novel technique for automated development of planar antennas. The presented methodology combines a flexible parameterization consisting of a resizable ground plane, relocatable discrete port, and elliptical-shaped patches and gaps, with the antenna topology being a Boolean transformation of its elementary building blocks. The complexity of the device’s architecture is controlled by the number of active patches/gaps, the position and dimensions of which are treated as continuous design variables. During the evolution stage, antenna topology is adjusted using computational intelligence methods, and it is further tuned using a gradient-based algorithm oriented toward improving the impedance matching over the frequency ranges of interest. The entire development procedure is unsupervised and exclusively driven by specifications. No human expert involvement is required whatsoever.

Our approach was extensively demonstrated by designing several antennas of different functionalities (narrow-band, broadband, dual-band, triple-band), all generated using identical algorithmic setups. The obtained geometries are highly unconventional. Numerical results were accompanied by prototyping and measurements of selected structures. Our framework may be considered a step towards antenna design automation. It offers a viable alternative to methods based on pixel antennas (due to improved flexibility of the antenna geometry) and free-form topology optimization (due to the global search capabilities). It can be used to develop antennas for demanding applications such as wearable or virtual reality devices and whenever a specific (small) physical space is allocated for the antenna.

The presented methodology can, in principle, be applied to other antennas (e.g., patch or horn). However, this would require the establishment of an antenna-type-specific parameterization without changing the underlying search engines. The parameterization must incorporate building blocks typical for the antenna type to be designed. In the case of patch antennas, most of the existing parameterization might be reused. For horn antennas, the building blocks should be of the 3-D type (various types of apertures and horn sections parameterized using splines, etc.). This will be considered as a part of future work.

## Data Availability

The datasets used and/or analyzed during the current study available from the corresponding author on reasonable request.
